# Single-cell multi-omics defines H3K27me3 remodelling in intervertebral disc degeneration with implications for regenerative intervention

**DOI:** 10.1038/s41413-026-00581-x

**Published:** 2026-09-07

**Authors:** Deepani W. Poramba-Liyanage, Xiaole Tong, Frank M. Riemers, Björn P. Meij, Yeter Dilek, Cindy G. J. Cleypool, S. Amir Kamali, Christine L. Le Maitre, Anne Camus, Danny Chan, Alexander van Oudenaarden, Peter Zeller, Marianna A. Tryfonidou

**Affiliations:** 1 Faculty of Veterinary Medicine, Department of Clinical Sciences, Utrecht University, Utrecht, The Netherlands; 2 Regenerative Medicine Center Utrecht, Utrecht, The Netherlands; 3 Department of Orthopedics, University Medical Center Utrecht, Utrecht, The Netherlands; 4 Department of Anatomy, University Medical Center Utrecht, Utrecht, The Netherlands; 5 Division of Clinical Sciences, School of Medicine and Population Health, University of Sheffield, Sheffield, UK; 6 Nantes University, Oniris, CHU Nantes, Inserm, Regenerative Medicine and Skeleton, RMeS, UMR 1229, Nantes, France; 7 School of Biomedical Sciences, The University of Hong Kong, Hong Kong SAR, China; 8 Oncode Institute, Hubrecht Institute-KNAW and University Medical Center Utrecht, Utrecht, The Netherlands; 9 Department of Molecular Biology and Genetics, Aarhus University, Aarhus, Denmark

**Keywords:** Bone, Pathogenesis

## Abstract

Utilizing key developmental cues and refining their orchestrating role in degeneration represents a promising strategy for understanding and treating intervertebral disc (IVD) degeneration, a major cause of chronic lower back pain. Here, we focus on notochordal cells (NCs), which originate from the embryonic notochord and reside in the developing nucleus pulposus. These distinctly vacuolated cells exhibit robust regenerative effects and hold promise for new therapeutic approaches. Dogs, like humans, suffer from the consequences of IVD degeneration. As the IVD matures and degenerates, NCs are replaced by smaller non-vacuolated NP cells (NPCs). The dog was employed as a model to capture, at the single-cell level, the heterogeneity of resident cells by studying the nucleus pulposus tissue at three stages (i.e., juvenile, young adult and degenerate adult). Here, we integrated transcriptomic data with repressive histone H3 lysine 27 trimethylation (H3K27me3) profiles at the single-cell level to assess changes in chromatin states and gene expression across this IVD degeneration-associated cell phenotypic transition. H3K27me3 enrichment on key genes involved in IVD development and homeostasis, such as *Brachyury* (*TBXT*), aligns with the observed attenuation during ageing and degeneration seen in both dog and human IVDs. This study further demonstrates that eliminating repressive histone marks, together with CRISPR-mediated gene transactivation, enhances *TBXT* gene expression in human NPCs derived from degenerated aged discs. Our findings underscore how extensive insights gained through single-cell omics can lead to the identification of crucial cellular cues that may enable degenerate NPCs to regain a healthier phenotype.

## Introduction

Developmental biology and regenerative medicine go hand in hand; the crosstalk between these two fields has allowed for new fundamental discoveries and improved therapeutic strategies. The use of notochordal cells (NCs) is considered to be a significant promise for an effective and long-lasting treatment of the degenerate and painful intervertebral disc (IVD)^1^ that has a projected 40 million sufferers worldwide. NCs reside only within the developing human IVD, and are characterized by their large, vacuolated appearance. Cell fate studies in mice have shown that with ageing and degeneration NCs transit to the smaller non-vacuolated nucleus pulposus cells (NPCs).^2^ NCs promptly dedifferentiate once cultured which makes them challenging to study and use in therapeutic approaches. Hence, a clear understanding of NCs’ biology and their transition into NPCs is of high relevance.

NCs play a pivotal role during development of the embryonic vertebral column.^3^ As the notochord regresses, ~10–18 weeks post-conception, the NCs persist and continue to play a role in IVD development.^4^ The fully formed IVD comprises three distinct components: the nucleus pulposus (NP),^5^ annulus fibrosus (AF), and cartilaginous end plates. The NP is the singular component derived from notochord,^6^ while the AF and cartilaginous end plates develop from the surrounding sclerotome.^7^ From 22 weeks post-conception in humans, the large vacuolated NCs gradually disappear and transition towards a more chondrogenic cell phenotype, the NPC.^8^ This cell type transition is completed around 10 years of age.^8^

While this transition is considered a maturation process, this phenomenon contributes also to early IVD degeneration that typically starts within the NP marked by reduction in glycosaminoglycan (GAGs). As IVD degeneration progresses, the AF can bulge and/or rupture with NP herniation into the spinal canal. This compresses nerves or the spinal cord, causing pain. In parallel, the degenerative extracellular matrix (ECM) changes permit ingrowth of blood vessels and nerves^9^ and following IVD rupture, immune infiltration exacerbates the inflammatory IVD microenvironment.^10^

Cell fate decisions within the IVD are molded by the embryonic origins, ongoing processes of maturation and by the inflammatory microenvironment associated with degeneration. Through single-cell sequencing, previous studies focused on cellular contributions and molecular mechanisms underlying IVD degeneration and often lacked the inclusion of NC-rich IVD tissue.^11–13^ We, therefore, studied cell fates during IVD maturation and early stages of IVD degeneration with a particular focus on the transition from NCs to non-vacuolated NPCs.

Humans lose their NCs early in childhood, making such research practically difficult and ethically burdensome. To overcome this limitation, we employed non-chondrodystrophic (NCD) dog breeds as a model. NCD dogs retain NC populations throughout much of their lifetime and experience IVD disease later in life. They exhibit similar pathophysiology and clinical representation, with natural IVD degeneration occurring in regions of higher load, including the lower lumbar and lumbosacral spine,^14,15^ as in humans. The clinical disease entity in NCD dogs is called degenerative lumbosacral stenosis and this unique model provides a valuable opportunity to investigate the NC phenotype throughout their lifespan, offering potential insights into regenerative approaches for IVD health.

We first characterized through single-cell RNA sequencing (scRNA-seq) using SORT-seq,^16^ distinct cell populations within the healthy NC-rich NP of NCD dogs, particularly as the cells mature, age, and succumb to disease in NP tissue from three groups (i.e., juvenile, young adult and degenerate adult). Then, to determine how chromatin states and expression profiles change during IVD ageing and degeneration, we studied, at the single-cell level, the transcriptome and distribution of the repressive histone H3 lysine 27 trimethyl (H3K27me3) modification in young adult and degenerate NP cells. H3K27 methylation and specifically H3K27me3 is a key marker of facultative heterochromatin mediating silencing of genes at different stages of development.^17^ As this is a process that is essential for cell fate specification and maintenance,^18^ the study focuses on H3K27me3 for investigating the epigenetic regulation of NC to NPC phenotypic transition. We further focused our analysis on key NC genes, such as *TBXT*, and studied the NC to NPC differentiation-dependent enrichment of H3K27me3 during ageing and degeneration. To translate these findings, as a proof of concept we investigated whether the epigenetic mark H3K27me3 silences *TBXT* gene expression in human NP cells derived from degenerated aged IVDs.

## Results

### Defining the major IVD cell types in NCD dogs

**Fig. 1:**
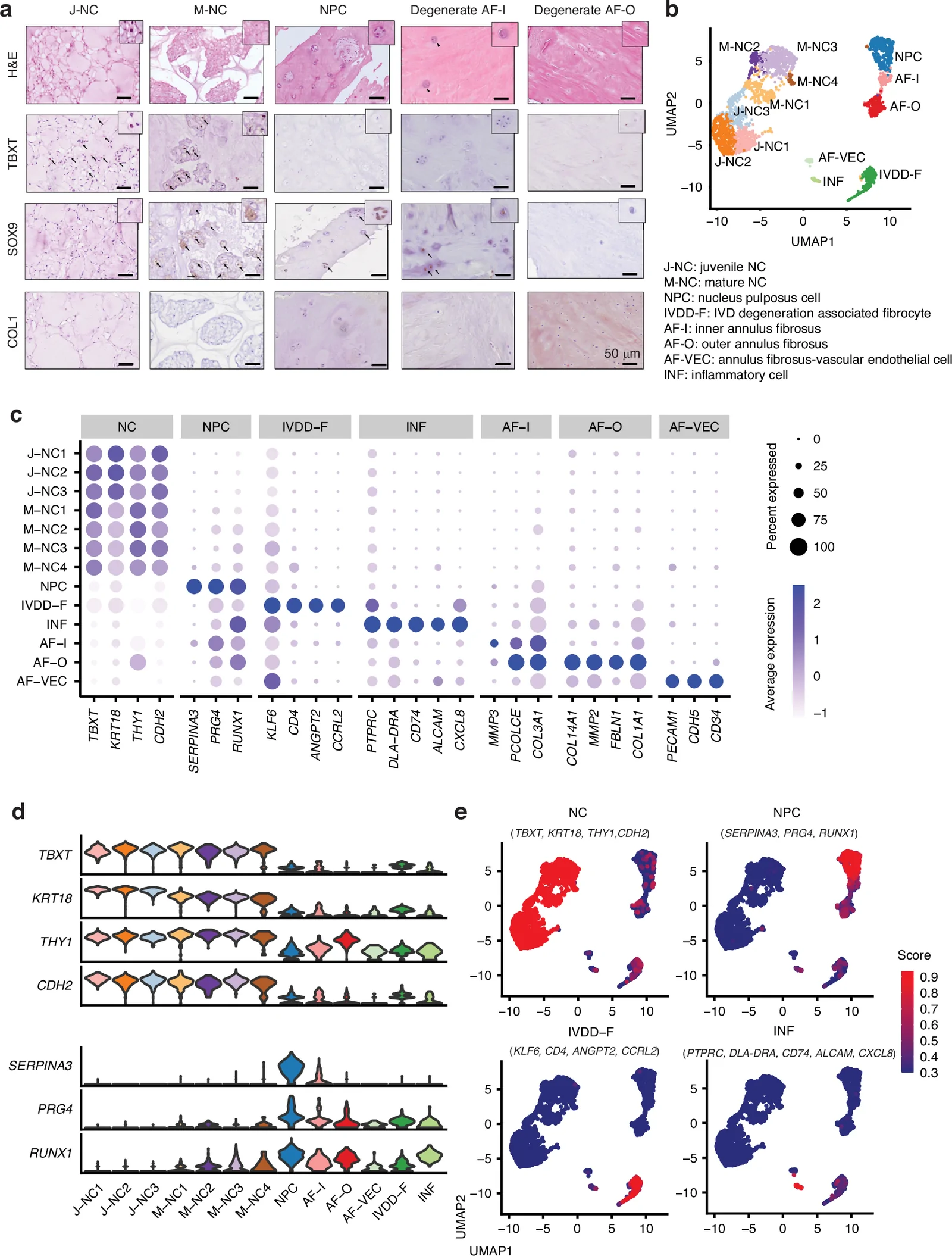
Defining the major IVD cell types in NCD dogs. **a** Representative examples of Hematoxylin and eosin (H&E)-stained sections and immunohistochemistry for TBXT, SOX9 and COL1 in normal juvenile and adult IVDs, and degenerate IVD tissue derived from dog patients suffering from low back pain. Cellular morphology and immunolocalization are shown in the magnification inserts; COL1 was detected only in the extracellular matrix. **b** Uniform manifold approximation and projection (UMAP) with unsupervised clustering. **c** Scaled dot plots of mean normalized expression for key marker genes per cluster; dot size indicates the %cells/cluster with detected expression. **d** Violin plots of NC and NPC cluster-specific genes (normalized expression). **e** Identification of key clusters by the average combined marker score using UCell. Juvenile NP: Harvested from stillborn NCD pups (*n* = 3; juvenile donors, NPs of the complete spine were pooled per donor) and Normal Adult NP: Harvested from 1 to 1.5-year-old healthy NCD dogs (*n* = 3; all thoracic and lumbar NPs were pooled per donor); both NPs are macroscopically demarcated from the AF and characterized by typical vacuolated NC organized in clusters within a GAG-rich ECM; Degenerate NP: Harvested from NCD patients undergoing standard-of-care discectomy for lumbosacral disc protrusion. These discs exhibit chondrogenic fibrocartilaginous metaplasia and a loss of vacuolated NCs (*n* = 3). Detailed donor information and classification criteria are provided in Tables S1–S3. Samples were further evaluated by immunohistochemical staining for TBXT, SOX9, and COL1 using DAB as the chromogen; arrows indicate positive cells. Scale bar: 50 µm

To characterize NP cell heterogeneity from health to degeneration, NP tissues were collected from stillborn dog IVDs (*n* = 3 stillborn donors) and normal adult dog IVDs (*n* = 3 adult donors), and from degenerate adult IVDs from dog patients undergoing standard-of-care discectomy of the protruding lumbosacral disc (*n* = 3 degenerate donors) (Tables S1–S3, Figs. 1a and S1a). To address the difficulty in discerning the AF and NP boundaries in patient tissues, the AF from one patient was also analysed (Figs. 1a and S1b, c). On histology, normal NP tissue showed typical vacuolated NC clusters dispersed in GAG-rich ECM and undetectable COL1 (Figs. 1a and S1a). The patient-derived tissue showed sparsely distributed cells surrounded only by pericellular GAG-rich ECM (Figs. 1a and S1a), consistent with commonly described histological degenerative changes.

Single cells were isolated per donor and subjected to SORT-seq, a scRNA-seq method that uses flow cytometry to sort cells into barcoded plates (Fig. S1d). A total of 2 597 cells were retrieved and sequenced, yielding 10 891 UMI/cell and 3 533 genes/cell. After normalization, scaling, and dimensionality reduction, clusters were visualized using UMAP (Fig. 1b) and annotated using commonly accepted marker genes for the different cell populations (Fig. 1c).

To test whether the donor source or the plate-based sequencing run influenced cluster formation, donor contribution was plotted for each cluster (Fig. S1e–g; Table S4). Only the AF vascular endothelial cell^19^ cluster came from the patient-AF; no cluster consisted of cells from a single donor or a single sequencing run. Three juvenile NC (J-NC 1-3) and four mature NC clusters (M-NC 1-4) were defined by *TBXT*, *KRT18*, *THY1*, and *CDH2*; a chondrogenic NPC cluster by *SERPINA3*, *PRG4*, and *RUNX1*; and an IVD degeneration-associated fibrocyte cluster (IVDD-F) by *KLF6*, *CD4*, *ANGPT2*, and *CCRL2* (Figs. 1b–e and S1h, i). These are illustrated by the average combined markers (Figs. 1e and S1i). Qualitative evaluation of tissue sections revealed comparable global patterns of TBXT expression across the three sample groups, with TBXT detected only in the juvenile and healthy adult NC-rich discs, but not in NPCs from degenerative samples (Fig. 1a).

We identified three AF clusters composed of AF-inner (AF-I), AF-outer (AF-O), and AF-vascular endothelial cell (AF-VEC),^20,21^ and a macrophage-dominated inflammatory cluster (INF) composed of cells from patient NP and AF expressing *PTPRC*, *DLA-DRA*, *CD74*, *ALCAM*, and *CXCL8* (Figs. 1b, c, e and S1h, i).^22,23^ The IVDD-F, AF-VEC, and INF clusters were exclusively derived from patient IVDs, indicating infiltration of inflammatory cells and vascular ingrowth (Fig. S1c, e–g; Table S4).

J-NCs were the dominant cell type in juvenile IVDs, and M-NCs were primarily derived from normal adult IVDs, whereas NPCs mainly made up degenerate adult IVDs (Fig. S1e–g; Table S4). The classical juvenile NP markers *TBXT*, *KRT18*, *THY1*, and *CDH2* were highly expressed in J-NCs and M-NCs, in contrast to mature NPCs and IVDD-F (Figs. 1c, d and S1h). Mature NPCs had higher expression of *SCRG1*, *SOX5*, and *COL2A1*, a pattern contrasting with M-NCs and IVDD-F (Fig. S1h).

The IVDD-F cluster formed a distinct population, possibly representing blood-derived fibrocytes or fibroblastic leukocytes, with features of both macrophages and fibroblasts.^24^ IVDD-Fs were identifiable by expression of *PTPRC* (*CD45*), *CD48*, *CD4*, *COL1A1*, *COL2A1*, and *CXCR4*, together with *ANGPT2*, *FN1*, *TBL1XR1*, and *SNAI1*.^25,26^ Interestingly, these cells lacked *CD34*, indicating a distinctive, tissue-resident fibrocyte population that maintained a pro-fibrotic transcriptional profile, including *KLF6* and *SPI1* (Figs. 1c, e and S1h).^25^ Notwithstanding, we cannot exclude that IVDD-Fs may also be partly derived and reflect the catabolic switch in NP and AF cells, as degenerate NPCs can express *CD4* and *CXCR4* alongside the common matrix genes *COL1A1* and *COL2A1*.^27,28^ In contrast, the INF cluster was indicative of tissue-resident immune cells,^25^ being *PTPRC* (*CD45*), *CD44*, and *CD74* positive and *CD34* and *CD4* negative, with high expression of the class II major histocompatibility (MHC-II) antigens *DLA-DRA* and *DLA-DQA1*, as well as *CXCR4*, *CXCL8*, *CCL7*, *RUNX1*, *NFKB1*, and *CTSS* (Figs. 1c, e and S1h).

The addition of patient-derived AF cells enabled clearer identification of AF cell subtypes. AF-I cells were distinguishable from NPCs by higher expression of *PCOLCE* and *COL3A1*, whereas NPCs expressed higher *SOX9* (Fig. S1h, i). AF-I cells were readily distinguishable from the AF-O cluster by high expression of *COL2A1*, *SOX5*, *SERPINA1*, and *ACAN*, marking a common feature of matrix metaplasia seen in AF-I degeneration.^15^ In contrast, AF-O cells showed high expression of *COL1A1*, *COL3A1*, *COL14*, *MMP2*, and *FBLN1*, and were identifiable by expression of *THY1* (*CD90*) and *CD55* (Fig. S1h, i).

### Exploring NC hierarchies across IVD ageing and degeneration

**Fig. 2:**
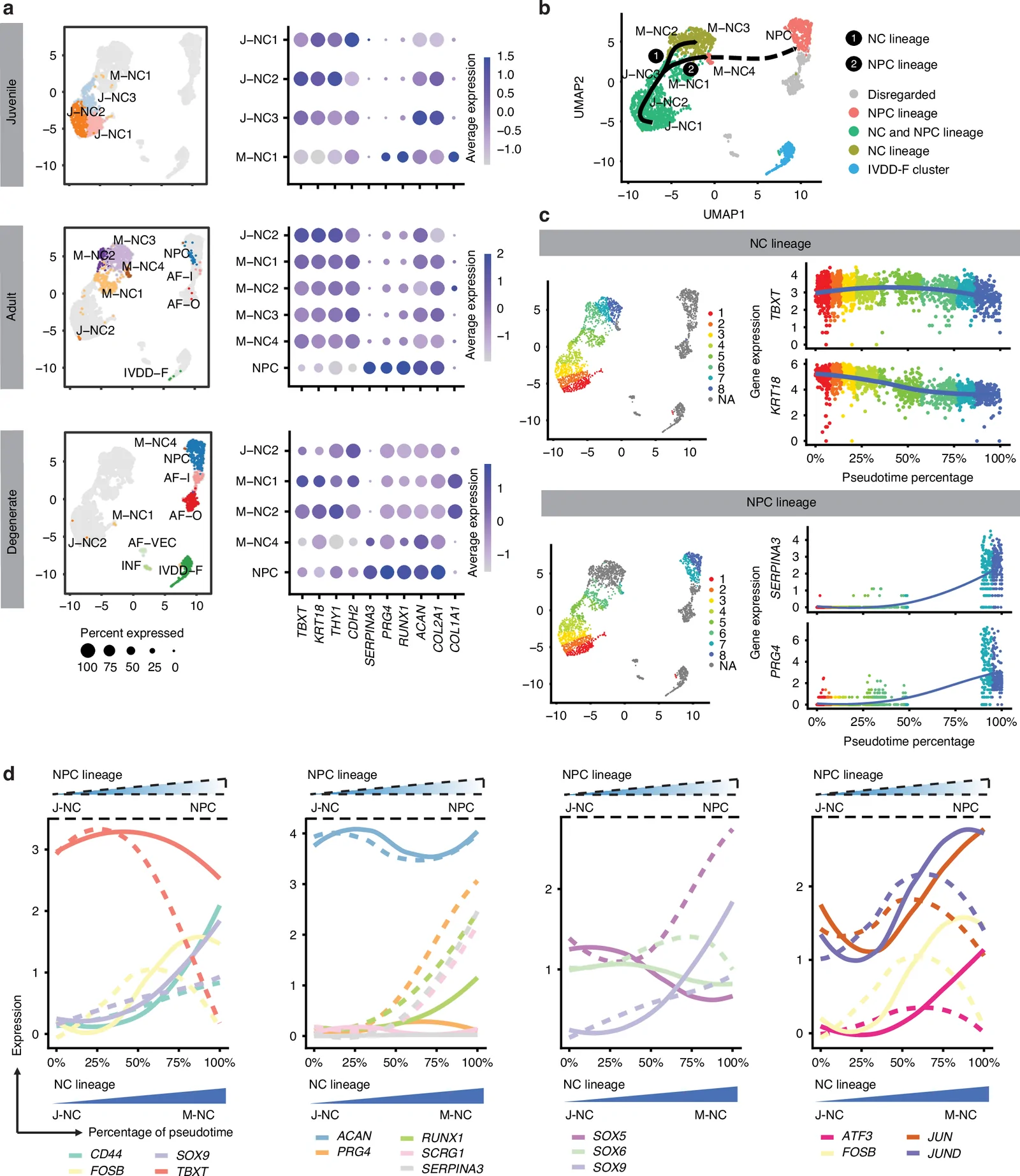
Exploring NC hierarchies across IVD ageing and degeneration. **a** UMAP highlighting the presence of NCs and NPCs in juvenile, adult and the patient-derived adult degenerate IVD. Scaled dot plots showing mean normalized expression of NC and NPC markers. Dot size indicates the %cells/cluster with detected expression. **b** Slingshot was run on UMAP clusters of NP origin using an Omega value of 1.5 ensuring a more accurate inference of cell lineages and developmental trajectories; AF-derived cells were excluded. The trajectories hypothesize the progression of the dominant NC lineage (solid line 1) and a second lineage (solid line 2). Extending the trajectory across the pseudotime gap reveals a possible trajectory from M-NC4 to NPC (dotted line). **c** UMAP of cells grouped into 8 pseudotime bins per lineage and fitted gene expression changes ordered along pseudotime. Two genes represent each lineage (NC lineage: *TBXT*, *KRT18*; NPC lineage: *SERPINA3*, *PRG4*). **d** Fitted gene expression curves along pseudo time comparing the NC lineage (solid line) and NPC lineage (dotted line)

We further explored age- and degeneration-dependent changes (Fig. 2a). Juvenile IVDs exhibited a notable enrichment for J-NC clusters (Table S4); the presence of M-NC1 cells suggested a transition to mature cells. In normal adult IVDs, M-NCs predominated, alongside a transition towards a more mature phenotype indicated by the presence of NPCs. Similar to human adult IVDs,^11^ patient-derived tissues contained limited NCs (Table S4). While all NCs from juvenile and normal adult sources expressed high levels of the key NC markers, NCs from patients exhibited ageing-related changes in the matrisome with high levels of *COL1A1* observed in J-NC2, M-NC1 and M-NC2 clusters (Fig. 2a). In contrast, patient-derived and normal adult disc-derived NPCs showed no striking differences in *ACAN* and *COL2A1* (Fig. 2a), indicative of a relatively unchanged population over ageing and moderate IVD degeneration. INF and IVDD-Fs were only present in tissues derived from dog patients, consistent with the presence of the tissue macrophage marker IBA-1 (Fig. S1a) and occasional populations of NPCs residing within a COL1-rich ECM on histology (Fig. S4i); with COL1 in the degenerate NP inconsistently present among patient samples (Fig. 1a). Altogether, this highlights similarities to human IVD degeneration, where immune infiltration contributes to the degenerative process,^10^ and NP cells produce cytokines and chemokines.^29^

The cellular hierarchy inferred cell lineages and pseudo-time analysis provided insights into the NP maturation process. All NP cells of notochordal origin,^2^ i.e., the 3 J-NC, 4 M-NC, NPC, and IVDD-F clusters were used in the unbiased reconstruction of differentiation trajectories; AF-derived cells were excluded using Slingshot’s cluster exclusion function.^30^ Pseudo-time analysis predicted two lineages: a NC lineage and a NPC lineage sharing 4 clusters: J-NC1, J-NC2, J-NC3, M-NC1 (Fig. 2b). Lineage inference suggested a branch point: NC lineage extended into M-NC2 and maturing M-NC3, while the NPC lineage progressed towards M-NC4 and NPC (Fig. 2b). The NPC lineage did not extend toward the IVDD-F lineage.

To examine our lineage hypothesis cells were grouped along the trajectory into 8 pseudo-time bins (Figs. 2c and S2a). The established NC and NPC markers along the defined pseudo-time axis supported the identified cell trajectories (Figs. 2c, d and S2a, b). Maturation of NCs was marked by sustained *TBXT* expression and an increase in *JUN, FOS* and *FOSB* (AP-1 transcription factor subunits with JUN) studied for its critical role in IVD development^31^ and degeneration.^32^ Their expression coincides with *CD44* expression, the receptor for hyaluronic acid, a distinctive non-sulphated GAG of the healthy IVD matrix^33^ in terminally differentiated NCs (Figs. 2d and S2b). Decreases in *TBXT* and *FOSB* indicated a transition to the NPC phenotype, accompanied by increased expression of *PRG4*, *RUNX1*, *SCRG1*, and *SERPINA3* (Figs. 2d and S2b). The study reveals that while both NCs and NPCs express high levels of *ACAN* and *COL2A1*, there were differences in the upstream SOX-trio transcription factors (TFs) *SOX5*, *SOX6*, and *SOX9* (Figs. 2d and S1g, S2b).^34^ The NC lineage was associated with an increase in *SOX9*, with *SOX5* and *SOX6* expression maintained at lower levels, whereas the NPC phenotype correlated with an increase in *SOX5* expression, further supporting the pivotal role of SOX genes within the IVD^35^ (Figs. 2d and S2b). In chondrogenesis, *SOX5* and *SOX6* have been reported to potentiate *SOX9* activity in driving *ACAN* and *COL2A1* expression.^34^ Qualitative analysis of tissue sections (Fig. 1a) confirmed the presence of SOX9 positive cells in NC-rich discs, as well as, in degenerate NPCs and AF-I cells, following the pattern of global gene expression seen in the three groups of samples (Fig. S1h).

### Defining the characteristics of NC phenotypes across cellular hierarchy

**Fig. 3:**
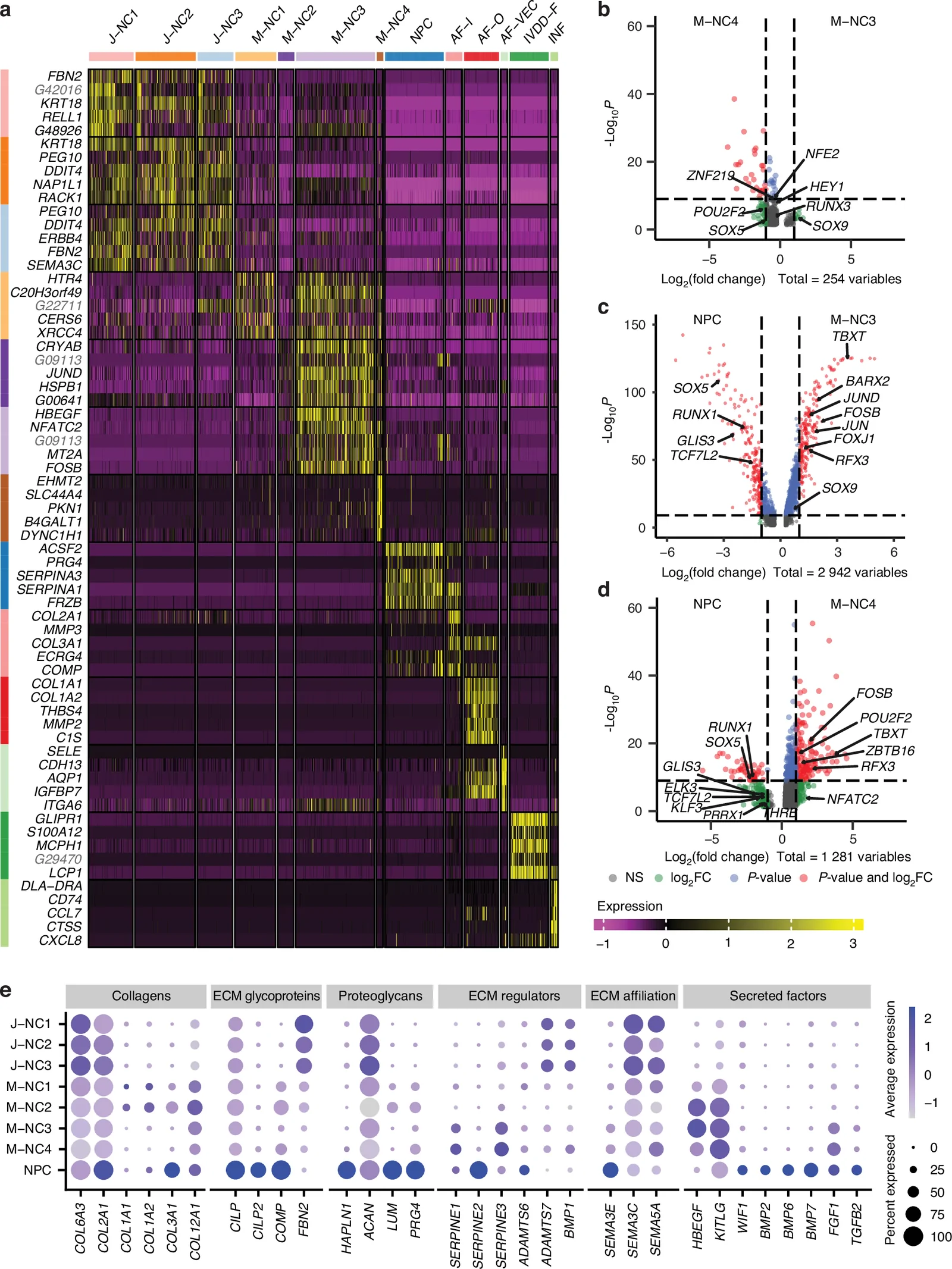
Defining NC phenotypes across cellular hierarchy. **a** Heatmap showing the top 5 differentially expressed genes (DEGs) per cell cluster ranked by average log fold change. ENSCAFG00000042016, ENSCAFG00000022711, ENSCAFG00000009113, and ENSCAFG00000029470 are abbreviated as G42016, G22711, G09113, and G29470, respectively. **b**–**d** Volcano plots showing differentially expressed transcription factors in the terminally differentiated NC (M-NC3 and M-NC4 cluster) and NPC (NPC cluster) populations. Red and green color: |log_2_FC| > 1; red are DEGs with a Benjamini-Hochberg adjusted *P*-value < 0.05. Gray: not significant in either; blue: *P* < 0.05 with |log_2_FC| < 1. **e** Scaled dot plot of key DEGs across six matrisome patterns focusing on NC and NPC populations

Two terminal trajectories were identified for NCs leading to the M-NC3 population, predominant in normal NC-rich discs, and the M-NC4 population bridging towards the NPC phenotype (Fig. 2b). In adult normal IVDs, M-NC3 and M-NC4 made up ~50% and ~5% of the cell population, respectively (Fig. S1f; Table S4). To characterize the signaling networks defining these terminally differentiated cell clusters and identify critical factors maintaining their states, their gene expression profiles were interrogated (Data File S1). A total of 1 825 differentially expressed genes (DEGs; log_2_FC > 1) were identified. The J-NC clusters shared many of the top DEGs while the M-NC clusters were distinct, suggesting possible phenotypic disparities, including TFs and matrisome genes related to the NC maturation process or ageing (Fig. 3a). In a targeted approach, surface marker profiles at the transcriptional level were not discriminative for the different cell clusters (Fig. S3a, b).

Differential TF expression was investigated using the SwissRegulon database. This database comprises 683 TFs, 509 of which were annotated in our dataset, and 75 DEGs. *TBXT*, *GLIS1*, *NR3C2*, *ID4*, *HIF1A*, *NR4A1*, *ATF4*, *NFATC2*, and *SOX9* (Fig. S3c), commonly present in all NC clusters have been reported before for the IVD or embryonic notochord in humans and mice.^1,20^ TF DEGs not previously associated with the IVD included *MZF1, CUX2, MAX, TFCP2, FOXJ1, RFX3, POU2F2, SOX13*, and *GLIS3* (Fig. S3c).

TF DEGs were also investigated between the terminal NC and NPC clusters (Fig. 3b–d). The key TF *SOX9*, was among the top differentially expressed TFs in M-NC3, along with *RFX3* and *FOXJ1*, downstream genes of *NOTO*. Both *RFX3* and *FOXJ1* are well documented in ciliogenesis, with NC being ciliated cells.^36^ *POU2F2* (*OCT2*) and *SOX13*, not previously associated with NCs, were preferentially expressed in M-NC4 (Fig. S3c). They are well studied in neuronal differentiation^37^ and cartilage biology, with *POU2F2* accelerating endochondral bone formation^38^ and *SOX13* being involved in cartilage progenitors.^38^ This supports our hypothesis, potentially linking M-NC4 to the chondrogenic-like phenotype of the NPC lineage.

The detected matrisome DEGs between NC and NPC clusters were categorized into collagens, glycoproteins, proteoglycans, ECM regulators, ECM affiliated, and secreted factors^39^ (Figs. 3e and S3d). NC clusters showed higher expression of *COL6A3* (a microfibrillar protein of the pericellular matrix), the proteoglycan *ACAN*, and semaphorins *SEMA3C* and *SEMA5A* (known for their anti-angiogenic and anti-neurogenic properties)^40^ (Fig. 3e). Conversely, NPCs expressed relatively more collagens (e.g., *COL2A1*, *COL3A1)* and the proteoglycans hyaluronan and proteoglycan link protein 1 (*HAPLN1*), lumican (*LUM*) and lubricin (*PRG4*) (Fig. 3e). HAPLN1, identified in NPC-rich IVDs of chondrodystrophic dogs,^41^ and LUM are related to degenerative changes in human IVDs.^42,43^ PRG4 detected at the gene^12^ and protein level^44^ has a yet undefined role in IVD degeneration. *SERPINE2*, an ECM modulator induced by pro-inflammatory stimuli to counteract matrix degradation via MMPs and ADAMTSs, was higher expressed in NPCs (Fig. 3e).^45^ Mature NC clusters expressed higher levels of secreted factors *HB-EGF* and KIT-ligand (*KITLG*) (Fig. 3e). Membrane-tethered HB-EGF interacts with EGFR for juxtacrine signaling,^46^ potentially maintaining NC clusters, which are notoriously challenging to dissociate into single cells during isolation. *KITLG*, vital for hematopoietic stem cell survival and self-renewal in the bone marrow niche,^47^ was elevated in NCs. Conversely, NPCs preferentially expressed *Wnt Inhibitor 1* (*WIF1*), shown to promote chondrogenesis,^48^ as well as growth factors *BMP2*, *BMP6*, *BMP7*, and *TGFβ2* known for their anabolic effects on NPCs.^49–52^

### Mapping repressing histone mark H3K27me3 at single cell resolution in NCs and NPCs

**Fig. 4:**
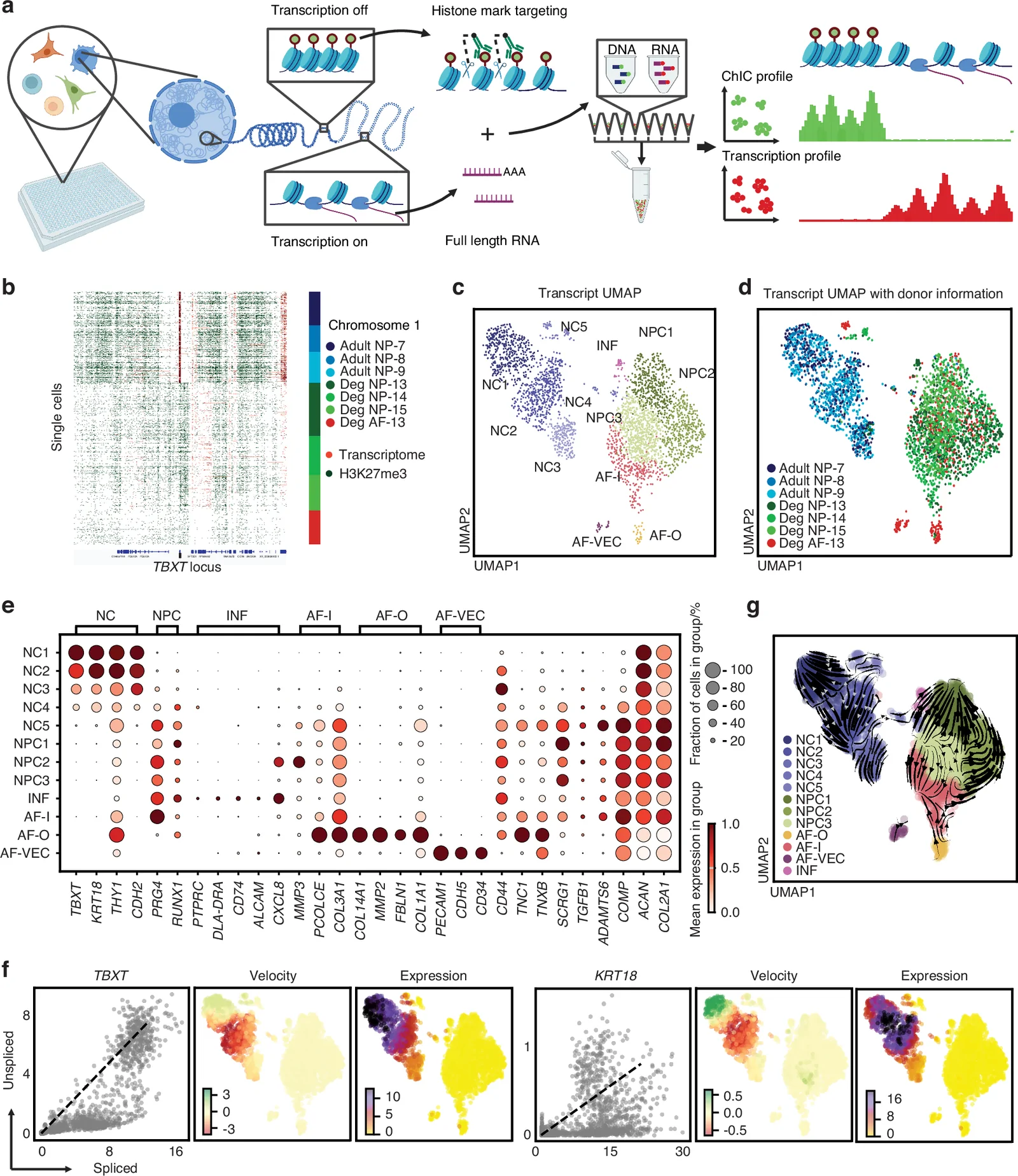
Application of single-cell T-ChIC to identify H3K27me3 dynamics in NC and NP cells. **a** Method schematic. **b** Single cell H3K27me3 enrichment (green) and transcript (red) along a 10 kb region of chromosome 1. **c** Full-length transcript-based UMAP, with 12 clusters identified based on unsupervised clustering. **d** Cells annotated by sample origin (*n* = 3 adult NC-rich and *n* = 3 degenerate discs obtained during standard-of-care surgery). The annulus fibrosus (AF sample) was from patient donor 13 (detailed donor information shown in Table S1). **e** Scaled dot plots of normalized DEGs per cluster. **f** *TBXT* and *KRT18* RNA splicing kinetics; positive velocity indicates gene upregulation (more unspliced mRNA) and negative velocity (more spliced RNA) indicates gene downregulation. **g** Visualization of RNA velocity. Cluster colors are specific to the single-cell T-ChIC data set and do not correspond to those used for the single cell RNAseq dataset presented through Figs. 1–3

How chromatin states underlie lineage specification in the maturing and degenerating IVD is poorly explored. To investigate the interaction between epigenetic H3K27me3 regulation and transcription at the single-cell level, we employed T-ChIC (Transcriptome + Chromatin ImmunoCleavage), which allows the detection of transcripts and histone modifications within the same cell using antibody targeted genome fragmentation as detailed in Blotenburg et al.^53^ To study the transition of M-NCs to NPCs, single cells were collected from normal and degenerate NPs of adult dogs (*n* = 3/group) (Figs. 4a and S4a). Specific transcript enrichment was observed, with H3K27me3 domains overlapping lowly transcribed and untranscribed regions, clearly separating normal and patient-derived cells (Fig. 4b).

The transcriptome UMAP consisted of 2 992 cells separating into 12 clusters (Fig. 4c). Using the marker genes used in the SORT-seq data set, 5 NC and 3 NPC clusters were annotated (Figs. 4c–e and S4e). Cells from healthy adult dogs mostly contributed to the NC clusters while patient-derived cells mostly contributed to the NPC clusters (Figs. 4d and S4f, g; Table S5). Overall, the cell types identified matched those observed in the SORT-Seq-based experiment (Figs. 1c, d and 4c–e). NC clusters expressed high *TBXT*, *KRT18, THY1, CDH2* and *ACAN* with NC3, the end differentiated cells, expressing the highest *CD44* (Fig. 4e). There was a gradual decrease of *TBXT* and *KRT18* expression in the NC clusters (Fig. 4e). Notably, NC4 and NC5 showed comparatively lower *TBXT* and *KRT18* levels (Fig. 4e). NC5 expressed the highest *TNC1*, *TNXB*, *PRG4*, *COL3A1* and *SCRG1* levels amongst the NC clusters (Fig. 4e), with many of these genes reported to have protective roles within the IVD. This may be indicative of an NC cluster contributing to the matrisome makeup of the adult disc.^1,12,20,54,55^ The 3 NPC clusters were chondrogenic with high expression of *COL2A1* and *COMP* but differed in *SCRG1*, *ADAMTS6* and *MMP3* (Fig. 4e; Data File S2). The expression of *SCRG1*, which is linked to the chondrogenic differentiation of MSCs, potentially indicates variations in matrix production among NPC clusters.^54^

An INF, AF-I, AF-O and AF-VEC cluster, all of patient origin, were identified (Fig. 4c–e). Notably, a subset of cells originating from patient AF was found within NC5 and NPC clusters (Table S5), potentially indicating cell contamination caused by NP tissue herniation, in line with the observed IVD degeneration grade. Histological sections of this patient revealed a few scattered vacuolated notochordal-like cells within a collagen-rich ECM, likely representing the transitional NC5 phenotype coinciding with non-vacuolated NPCs, both residing with a COL1 immuno-positive ECM (Fig. S4h, i).

The full-length transcript recovered as part of T-ChIC facilitates the retrieval of immature/unspliced RNA allowing for evaluation of the cell state (Figs. 4f and S4j). To assess the directionality of differentiation within the NC clusters, and to assess if NCs gradually differentiated towards an NPC phenotype, mRNA splicing kinetics were analyzed and velocities projected (Fig. 4f, g). For both *TBXT* and *KRT18*, a reduction was observed in unspliced (actively transcribed) reads from NC1 to NC3, indicating that these genes are being turned off (Fig. 4f). This implies directionality from NC1 to the CD44^+^ NC3 cluster. These findings highlight dynamic cellular transitions within the IVD, with the NC3 cell stage representing an intermediate step where cells begin to suppress the expression of *TBXT* and *KRT18*.

### Differential H3K27me3 enrichment reveals epigenetic regulation of NC-to-NPC fate transition

**Fig. 5:**
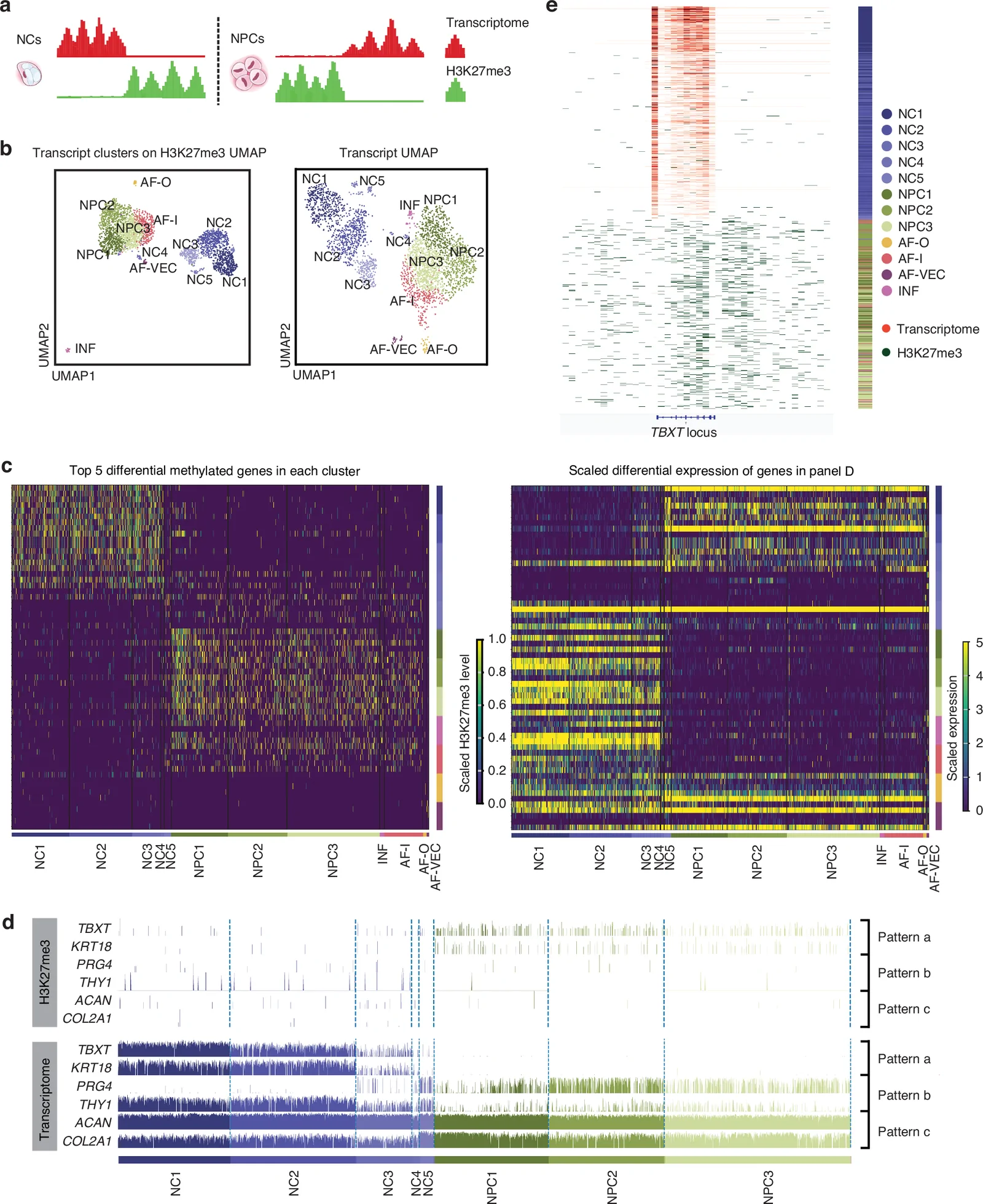
Differential H3K27me3 enrichment reveals epigenetic regulation of the NC-to-NPC fate transition. **a** Co-mapping of gene expression and H3K27me3 revealing cell-type-specific patterns. **b** H3K27me3 UMAP colored by the 12 identified transcript-based cell clusters. They largely overlap with the transcript-based clusters, indicating that cell states are principally reflected by H3K27me3. **c** Heatmaps of the top 5 differentially methylated genes per cluster, sorted by average log fold change, with corresponding scaled expression, highlighting distinct patterns of H3K27me3 and gene expression defining NC and non-NC cell populations. Cluster colors are specific to the single-cell T-ChIC data set and do not correspond to those used for the single cell RNAseq dataset presented through Figs. 1–3. The complete heatmaps, including gene names, are shown in Fig. S5c. **d** Full-length RNA and H3K27me3 track plots revealing three main patterns: (a) H3K27me3 enrichment with gene silencing, (b) differential expression independent of H3K27me3, and (c) expressed genes without H3K27me3-mediated silencing. **e** Genome browser view of the TBXT region (1 kb region; chromosome 1) showing an H3K27me3 domain gained (green) accompanied by loss of *TBXT* expression during the NC-to-NPC transition

Chromatin modifications were analyzed relative to gene transcription to assess H3K27me3 enrichment and its role in lineage specialization during the NC-to-NPC transition. Fragment counts within a ± 50 kb window around transcription starts sites after dimensionality reduction and UMAP, revealed 11 distinct clusters providing a cell-specific H3K27me3 landscape (Figs. 5a, b and S5a, b). Cells were color-coded by transcriptome-inferred cell types (Figs. 5b and S5a) revealed strong global agreement between H3K27me3-based and transcript-based UMAPs, indicating that H3K27me3 largely distinguishes cell states. This was reinforced by the heatmap of the top five differentially methylated genes per cluster (Figs. 5c and S5c left), their scaled differential expression (Figs. 5c and S5c right), and the track plots of representative loci highlighting distinct H3K27me3 and transcriptional patterns between NC and non-NC populations (Figs. 5d and S5d).

To further characterize how H3K27me3 contributes to transcriptional regulation in the IVD, we focused on key TF and matrisome genes (Figs. 5c, d and S5c, d). Three main different patterns were observed: (a) H3K27me3 enrichment in cell types not expressing the gene, indicating silencing (e.g., *TBXT*, *KRT18*); (b) differential expression without corresponding H3K27me3 enrichment, suggesting regulation independent of polycomb repression (e.g., *THY1* and *PRG4*); and (c) ubiquitously expressed genes not acquiring a silent chromatin state throughout the cell clusters (Figs. 5d and S5d).

Focusing on differences between NCs and NPCs, *TBXT, KRT18, CDH2* and *SHH* exhibited Pattern-a in NPCs with notable H3K27me3 enrichment, while these loci showed minimal H3K27me3 in NCs actively expressing the genes (Figs. 5d and S5d, e) as exemplified in Fig. 5e for the *TBXT* locus at the single cell level. Interestingly, in NC3 cells, *TBXT* expression was reduced with increased H3K27me3 coverage, accompanied by an upregulation of *GLI2* (Figs. 5c and S5c, d). *KRT18*, *PRG4*, *KRT3* and *THY1* also showed reduced expression in NC3 cells but without distinguishable H3K27me3 coverage (Figs. 5d and S5d, e), which may reflect either direct regulation by the TBXT regulome (e.g., KRT18) or downstream effects of broader transcriptional changes during the NC-to-NPC transition.^56^ This observation supports the potential trajectory from NCs to NPCs, with NC3 representing an intermediate stage where cells begin to suppress *TBXT* and other key regenerative NC markers.

Among the top genes not previously associated with NCs, but showing high differential H3K27me3 mediated transcriptional repression compared to NPCs, were genes (e.g., *GABBR1* and *NCAM1*) potentially associated with the NC matrisome (Figs. 5c and S5c, d, Data File S2, S3). While transcriptome analysis captured major cell-to-cell differences, H3K27me3 enrichment uncovers intermediate differentiation stages and potential cell fate trajectories. This study showcases the promise of T-ChIC, using chromatin profiling to identify transitional phenotypes and regulatory landscapes otherwise masked in steady-state transcriptomes.

### Methyl transferase inhibition of human NPCs to reverse their degenerative phenotype

*TBXT* gene expression in bulk analysis is detectable only in a subset of degenerate and aged NP tissues,^57^ with a limited number of cells expressing robust *TBXT* at the single cell level.^12^ At the protein level it decreases in human degenerated and aged IVDs^58,59^ and dog discs (Fig. 1a). Based on the observation of H3K27me3 accumulation at the silenced *TBXT* locus during NC-to-NPC transition (Figs. 5d, e and S5d, e), we hypothesized that reducing the H3K27me3-dependent epigenetic barrier with GSK126 would allow re-expression of *TBXT*. Polycomb-repressive complex 2 (PRC2) is the only known mammalian methyltransferase that catalyzes H3K27 methylation;^60^ its EZH2 catalytic subunit can be chemically disrupted by GSK126.^61^

**Fig. 6:**
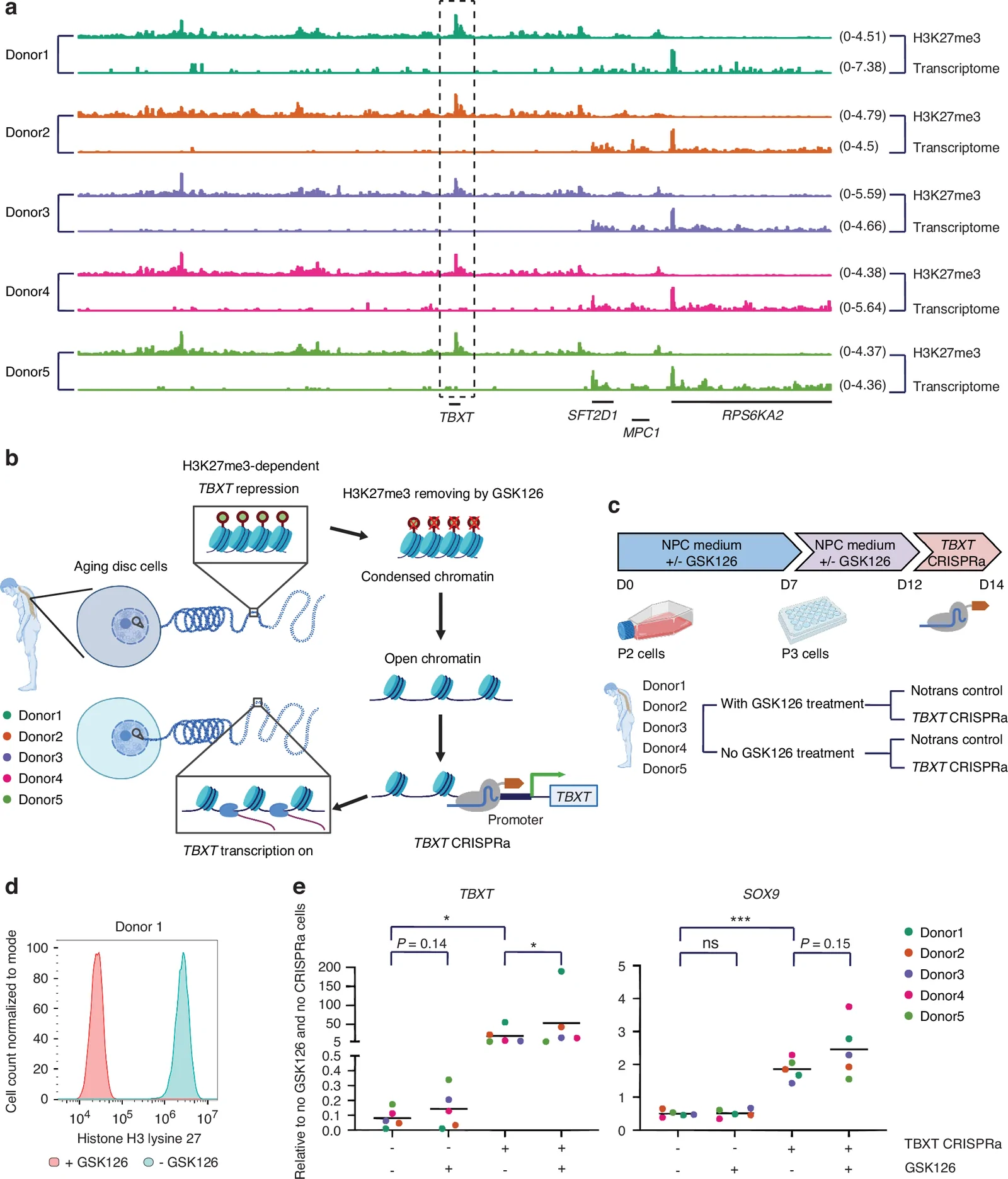
Demethylase treatment of human NPCs to reverse their degenerative phenotype. **a** T-ChIC profiles from five aged human NPC donors show consistent H3K27me3 enrichment at the silent *TBXT* locus. **b** Schematic of the demethylation by GSK126 inhibitor and CRISPR activation (CRISPRa) of TBXT. **c** Experimental setup. **d** Representative flow cytometry showing H3K27me3 loss after GSK126 treatment. **e** *TBXT* and *SOX9* expression following GSK126 treatment and combined GSK126 + CRISPRa; *P < 0.05; ***P < 0.001

To evaluate whether this silencing is also linked to H3K27me3 in human NPCs, and to determine the relevance of our dog single-cell findings, we applied T-ChIC profiling to P3 cultured NPCs of five primary human donors aged 58–89 years (Figs. 6a and S6a–d; Data File S4, S5). In all five donors, *TBXT* remained transcriptionally silent and exhibited local H3K27me3 enrichment (Figs. 6a and S6a), consistent with the epigenetic repression observed in dog NPCs. Notably, the epigenetic-transcriptional patterns we previously categorized in the dog dataset of NPCs (Figs. 5e and S5d, e)—particularly Pattern-a (H3K27me3 enrichment with transcriptional silencing), as seen in *TBXT*, *SHH*, and *GLI2*—were conserved in the human profiles (Fig. S6a). While other patterns showed more variability across species and donors, this conserved repression pattern strengthens the translational relevance of our dog findings.

To assess the effects of H3K27me3 removal on *TBXT* expression, the five human NPCs were treated with GSK126 (Fig. 6b, c). After 2 weeks, global H3K27me3 levels were reduced (Figs. 6d and S6e) leading to a geomean 1.43-fold induction in *TBXT* expression (*P* = 0.138; Fig. 6e). The expression of common NPC genes - including *SOX9*, *KRT18*, *ACAN*, *COL1A1*, with modest to absent H3K27me3 marks and the relatively hypermethylated *COL2A1*, remained stable (Fig. S6f). This suggests that their transcriptional regulation may not be strongly dependent on the repressive H3K27me3 mark under these conditions.

Removal of repressive chromatin alone may be insufficient to fully reactivate silenced genes in aged and degenerate human NPCs lacking the necessary transcriptional machinery to initiate expression from these heterochromatic regions. To overcome this barrier, we employed the dCas9–SAM transactivation system to recruit activators and remodel chromatin at the *TBXT* promoter. This approach (Fig. 6b, c) significantly increased *TBXT* expression across all donors, with a geomean 231-fold induction (*P* = 0.011; Fig. 6e). To further enhance *TBXT* levels, we combined CRISPRa with PRC2 inhibition using GSK126. This approach resulted in a geomean 1.9-fold increase in *TBXT* expression compared to CRISPRa alone (*P* = 0.032), indicating the synergistic activation effect (Fig. 6e). Higher *TBXT* levels due to CRISPRa correlated with higher *SOX9* expression, with the GSK-synergistic approach trending to be 1.28-fold geomean higher compared to CRISPRa alone (*P* = 0.145), possibly reflecting an early downstream effect of elevated *TBXT* levels on the transcriptional program. Within this overly short time frame to assess effects at the matrix level, *ACAN*, which is downstream of both TBXT and SOX9,^62^ remained at baseline levels 48 h following CRISPRa, while *COL2A1* and *COL1A1* expression significantly decreased (Fig. S6f, respectively, *P* = 0.07 and *P* < 0.001). Altogether, combining epigenome editing and transcriptional engineering demonstrated that removing the H3K27me3 barrier enhanced reactivation of the decommissioned *TBXT* gene in aged and degenerate human NPCs.

## Discussion

**Fig. 7:**
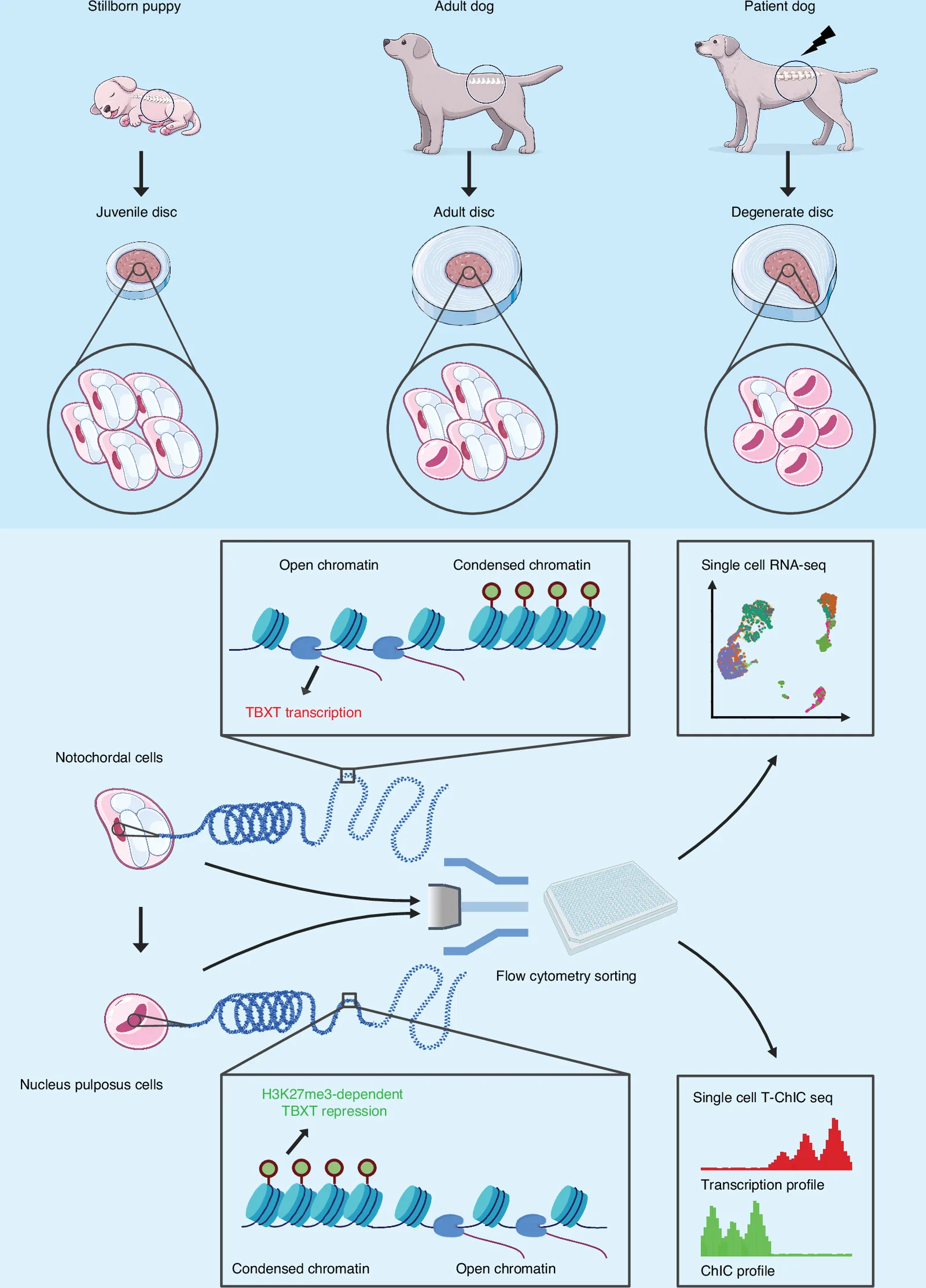
Schematic representation of the proposed transition from large, vacuolated notochordal cells to non-vacuolated cells in the nucleus pulposus tissue derived from non-chondrodystrophic dogs at distinct life stages. The retrieved single-cell transcriptome and H3K27me3 atlas, along with follow-up proof-of-concept studies, focused on epigenetic modulation of the *TBXT* gene involved in this transition. Proof-of-concept data demonstrating H3K27me3 removal by GSK126 in human aging NPCs suggest translational potential to partially reverse NPCs’ degenerative phenotype through epigenetically facilitated cell fate change

Here, we present the first single-cell transcriptomic and H3K27me3 atlas of NC-rich discs from NCD dogs (Fig. 7) that also exhibit back pain associated with natural lumbosacral IVD degeneration and NC loss, diagnosed and treated as in humans.^14^ Unlike chondrodystrophic breeds with distinct genetic drivers (*FGFR4*),^63–65^ NCD breeds may provide a representative landscape of the complex, age-related epigenetic remodeling observed in human patients. In a comparative epigenetic assessment using transcriptome–chromatin profiling of aged human NPCs, we demonstrate conservation of H3K27me3-mediated silencing of key NC genes and reinforce the NCD model as a high-fidelity surrogate for human disc research.

The SORT-seq and T-ChIC seq plate-based sequencing platforms enabled handling of larger NCs and offered a modular design suited to unpredictable sample availability from the clinic. Although modest cell counts may underrepresent rare cell types, these platforms provided high sequencing depth and sensitivity to identify subpopulations during the NC to NPC transition and facilitated integrative transcriptomic-epigenomic analysis. SORT-seq retrieved 1 646 NCs, and T-ChIC retrieved 1 256 NCs, from a total of 2 597 and 3 377 single cells sequenced, respectively. As expected, most NC populations were found in juvenile and normal adult IVDs, while patient IVDs also retained some NC populations, confirming that the transition stages from NCs to NPCs were captured within the sample groups. By prioritizing ethical access to dog samples, we utilized two healthy life stages (juvenile and young adult) and naturally occurring disc disease rather than a continuous aging spectrum, and hence cannot exclude that rare cell types or transient regulatory dynamics may be underrepresented. Nonetheless, the design captured a broad spectrum of NC differentiation and phenotypic changes associated with maturation and degeneration that is difficult to study in human embryos and children, where NC retrieval is ethically burdened and limited. Showing that the NC clusters align with the general marker profiles of previously identified handfuls of NCs in human studies,^11,20^ we can now further address the vast variety of NC phenotypes.

Reconstruction of the NC hierarchy at the transcriptional level supports the existence of two terminal NC populations. The M-NC3 population, with high *CD44* levels, made up most healthy NC-rich discs. This builds on the work of McCann et al., whose lineage-tracing studies in Noto-Cre mice showed that embryonic NCs act as tissue-specific progenitors within the IVD and, during aging and degeneration, transition to smaller, non-vacuolated NPCs.^2^ Jiang et al.^11^ also identified CD44^+^ cells as NCs in the human adult disc; hence, the present work reinforces the concept that these CD44^+^ cells are NC remnants in the adult dog disc. In addition, *TBXT* was highly expressed in NC clusters compared to mature NPCs, consistent with Gan et al., who also used *TBXT* as an NC marker,^12^ and is corroborated by detection of TBXT in juvenile and healthy adult dog NC-rich discs. Furthermore, our data suggest that these precursor cells further differentiate, evolving from NCs in the early IVD to NPCs in the adult and degenerated IVD, with the M-NC4 population bridging toward the NPC phenotype. In the NC-rich adult dog disc, M-NC4 cells comprised ~5% of the population, and their co-occurrence with NPCs in both normal adult and NPC-rich patient IVDs support a model in which the M-NC4 cells represent intermediate states along a gradual NC-to-NPC differentiation trajectory.

Focusing on the NC-to-NPC transition as part of the maturation process and progression toward degeneration, we used the newly developed single-cell T-ChIC^53^ to obtain an unbiased, single-cell-resolution view of H3K27me3 enrichment, a repressive chromatin barrier, together with full-length RNA in NP cell types from adult and patient-derived discs. We demonstrate that H3K27me3 enrichment at key genes (e.g., TBXT, KRT18, CDH2, and SHH) occurs as NCs differentiate into NPCs, indicating distinct epigenetic changes associated with this shift in transcriptional phenotype (Fig. 7). The enrichment pattern further supports a possible trajectory from NCs to NPCs, with intermediate cell stages that suppress TBXT and other key NC markers. This is further supported by RNA velocity analysis, which showed a progressive reduction in unspliced mRNA for these genes across the NC clusters, consistent with transcriptional silencing during NC maturation and the NC-to-NPC transition. Within this context, illustrative examples of novel genes actively transcribed in NCs but selectively repressed in NPCs included the gamma-aminobutyric acid type B receptor subunit 1 (*GABBR1*), involved in GABBA signaling with roles in neurotransmission, regulation of hematopoietic progenitor cells, and immunomodulation,^66^ and yet uncharacterized roles in NCs; and *NCAM1*, a neural cell adhesion molecule crucial for cell-to-cell and cell-matrix interactions during development.^67^ As these genes were also found to be repressed in aged human NPCs in this study, it is tempting to hypothesize that aging reinforces or maintains the repressive chromatin landscape, contributing to the loss of NC-like features over time.

TBXT is expressed in human IVD throughout life but declines with aging and degeneration.^12,58^ It has been shown to bind the promoter region of *ACAN*^62^ and directly activate SMAD3 signaling in human NPCs, mediating a matrix-anabolic effect at the tissue level.^68^ In this context, the present study shows increased H3K27me3 levels at the *TBXT* locus in degenerated dog and human NPCs, reinforcing its role as an epigenetically silenced regenerative marker in NPCs. This was supported by functional assays in human NPCs demonstrating that removing the H3K27me3 barrier with GSK126 reactivated *TBXT* expression. However, because transcriptional reprogramming may require more than chromatin derepression, particularly in aged human NPCs lacking appropriate transcriptional activators, we targeted the *TBXT* promoter using dCas9-SAM to induce *TBXT* expression. Our strategy successfully reactivated *TBXT* and its downstream target *SOX9*, confirming that removing the H3K27me3 ‘brake’ allows more efficient gene re-expression. These TFs target specific enhancer regions within matrix-associated genes, e.g., *ACAN* and *COL2A1*.^62,69^ However, this gene re-expression did not translate into functional matrix synthesis. This discrepancy is likely twofold: first, the 2D monolayer environment and short-term culture conditions do not favor the phenotypic capacity of NPCs to assemble ECM; second, while we successfully modulated one epigenetic layer (H3K27me3), additional repressive factors at these loci may still limit the magnitude of activation. Further studies are needed to confirm that re-emergence of NC markers is associated with improved matrix synthesis and to determine whether these effects would be even more robust in less-aged human NPCs.

Interest in epigenetic regulation in IVD degeneration and regeneration is increasing,^70,71^ and the observed biological effects are context-dependent. Recently, it was shown that loss of histone deacetylase SIRT6 promotes cell senescence and accelerates age-associated IVD degeneration in mice with conditional deletion of SIRT6 in the disc.^72^ Several studies report that EZH2, the methyltransferase responsible for H3K27 trimethylation, is upregulated^73,74^ or downregulated^75^ in human degenerative IVDs. By modulating H3K27me3 levels at target gene promoters, EZH2 either suppresses or activates gene expression, respectively, and promotes NP cell senescence-associated inflammation^75^ and accelerates ECM degradation.^74^ Inhibition of EZH2 restores SOX9 expression and cartilage-related genes, alleviating endplate and NP degeneration.^74^ Moreover, EZH2-mediated silencing of regulatory factors (e.g., DDX1, DKK1, NOX-4, or miR-129-5p) activates inflammatory and pyroptotic pathways.^73,75–78^

The present study, including the abovementioned studies targeting EZH2 modulation, carries a caveat: global demethylation does not selectively target the gene of interest, e.g., the *TBXT* locus in the present study, but will broadly alter the epigenetic landscape, potentially disrupting essential regulatory networks. Achieving precise and safe therapeutic epigenetic modulation requires refinement that accounts for patient variability and the fact that the epigenetic landscape also depends on disease stage. Future translational efforts using in vivo CRISPR-dCas9-based systems^79^ should focus on developing gene-specific epigenetic editing approaches that enable programmable, locus-defined modification.^80^ The present study contributes to this largely unexplored field. It offers a multi-omics-based pipeline to assess histone modifications, deepen understanding of disease endotypes, and enable the discovery of key cellular transitions that can be harnessed for regenerative approaches to degenerate IVD.

## Materials and methods

### Tissue collection and stratification on degeneration

NP tissue was dissected from the IVD of NCD dogs: (a) 3 stillborn puppies with informed consent from the owner; (b) 3 healthy adult experimental dogs euthanized for unrelated studies, and (c) from 3 dog patients with lumbosacral degenerative disc disease confirmed on T2-weighted MRI and scheduled for standard-of-care discectomy surgeries with consent from the owner. A single AF tissue dissected from dog patients was included in each single-cell RNA and T-ChIC analysis to inform transcriptomic profiling of AF cells.

Human NPCs were isolated from five donors (Table S6). Informed consent was obtained during life, allowing the use of these bodies for educational and research purposes through the body donation program to the Anatomy Department of the University Medical Center Utrecht, the Netherlands.

### Single-cell RNA sequencing

Single-cell RNA sequencing was performed according to an adapted version of the SORT-seq protocol^16^ by Single Cell Discoveries (Utrecht, NL). SORT-Seq accommodates the large size of vacuolated NCs (~40 µm ∅), which can exceed the limits of standard microfluidic droplet-based platforms (e.g., 10× Genomics), offered flexibility by allowing plate storage prior to library preparation and provided for high sequencing depth and sensitivity. To minimise batch-associated variation, all plates were processed in parallel through cDNA synthesis, library preparation, and sequencing. Reads were demultiplexed, filtered, mapped to the canine genome (CanFam3.1) and counted. Cells expressing < 500 or > 7 500 genes, < 30 000 Unique Molecular Identifier counts, and > 10% mitochondrial genes were filtered out. Next, data were normalised and scaled and PCA was used for dimension reduction, followed by clustering analysis. UMAP visualization showed no clustering by plate or condition (Fig. S1g); therefore, no batch correction or dataset integration was required. DEGs between clusters were obtained using a parametric Wilcoxon rank sum test (*P* < 0.05). Pseudo-time analysis was performed with the Slingshot package,^16^ for the unbiased reconstruction of differentiation trajectories. Clusters of NC and NPC origin were used in the analysis, with AF-derived cells of sclerotome origin excluded using Slingshot’s cluster exclusion function. Slingshot was then run on the UMAP.

### Single-cell T-ChIC

We employed T-ChIC for the detection of full-length transcripts and histone modifications within the same cell, using a plate-based method for downstream processing as detailed in Blotenburg et al.^53^ (Fig. S4a–d) and modified for small primary cell samples. Transcript library preparation entailed RNA fragmentation, repair, poly-A tailing, reverse transcription. ChIC library preparation included DNA repair, A-tailing, adapter ligation, second strand synthesis purification, in vitro transcription, rRNA depletion, and further RNA processing to prepare the sequencing libraries. Sequencing of both libraries was performed using paired-end 100 bp reads, and transcriptome and ChIC analysis including barcode-based demultiplexing, trimming, mapping, to obtain Fastq, .csv and .loom files using the T-ChIC Snakemake. Further processing included cell filtering and normalization, leading to PCA, UMAP, and pseudotime calculations.

### H3K27me3 removal and CRISPR-mediated activation of TBXT in human NPCs

To study the effect of H3K27me3 removal in human NPCs, 1 million P1 cells (Table S6) were equally seeded in two T75 culture flasks and cultured with expansion medium (hgDMEM; 2.2 mg/mL NaCl (Sigma-Aldrich); 10% FBS (Biowest); 1% Penicillin/Streptomycin) alone or supplemented with 500 nmol/L GSK126 (EZH2 inhibitor, Sigma Aldrich). After 1-week expansion (37 °C, 5% CO_2_, 20% O_2_) NPCs were re-seeded in expansion medium in 24-well plates (20 000 cells/cm^2^). After 5 days, CRISPRa was used to activate *TBXT* transcription using a gRNA targeting vector designed to target the promotor region of the *TBXT* gene using p2T-gRNA (template from addgene, #71485) vector backbone. The gRNA targeting vector was co-transfected with PB-SAM-Dest vector (addgene, #102563) to achieve transcriptional activation at the *TBXT* promoter.^81^ Transfection was carried out using Lipofectamine3000 (Invitrogen) for 24 h, and cells were collected for RT-qPCR 48 h after transfection.

## Supplementary information

Supplementary file

## Data Availability

All data needed to evaluate the conclusions in the paper are present in the paper and the Supplementary Materials. Raw RNA seq data is available through the GEO accession number (GSE284399). Raw T-ChIC seq data is available through the GEO accession number (GSE285151). Bulk human T-ChIC seq data is available through GEO accession number (GSE300462). The processed data is available for download on ZENODO (https://doi.org/10.5281/zenodo.19450740).
