# Supplementary material for: Single-cell multi-omics defines H3K27me3 remodelling in intervertebral disc degeneration with implications for regenerative intervention
Source: Bone Res. 2026 Sep 7;14:97. doi: 10.1038/s41413-026-00581-x (PMC13550594; doi:10.1038/s41413-026-00581-x)
Supplement: Supplementary file 1 [file 41413_2026_581_MOESM1_ESM.pdf]

Title: Single-cell multi-omics defines H3K27me3 remodelling in intervertebral disc degeneration with implications for regenerative intervention

List of Supplementary Materials

Supplementary Materials and Methods
Fig. S1. Defining major IVD cell types.
Fig. S2. Exploring NCs hierarchies across disc ageing and degeneration.
Fig. S3. Identification of NCs subpopulations within the IVD.
Fig. S4. Application of single-cell T-ChIC for transcriptome and H3K27me3 dynamics in NCs and NPCs
Fig. S5. Differential H3K27me3 enrichment within disc cells unravels insights into epigenetic regulation of NC to NPC fate transition.
Fig. S6. Demethylase treatment of human NPCs to reverse their degenerative phenotype.

Table S1: Sample details and grading of dog discs used in the study
Table S2: Breakdown of the parameters used for scoring degeneration of NP
Table S3: Breakdown of the parameters used for scoring degeneration of AF
Table S4: Cells per cluster per tissue type and per cell donor in the SORT-Seq data set
Table S5: Cells per cluster per tissue type and per cell donor in the T-ChIC data set
Table S6: Donor information
Table S7: List of qPCR primers
Table S8: Immunohistochemical staining

<u>Data files S1-S5 are provided in an excel format in zenodo (DOI: 10.5281/zenodo.19450740)</u>

Data file S1: Dog_differential expression_SORTseq
Data file S2: Dog_differential expression_T-ChIC
Data file S3: Dog_differential methylation_T-ChIC
Data file S4: Human_transcriptome_bulk T-ChIC
Data file S5: Human_H3K27me3_bulk T-ChIC

Supplementary Materials and Methods

1. Dog sample collection and stratification on degeneration

Healthy dog (large breed dogs; non-chondrodystrophic; *Canis lupus familiaris*) intervertebral discs (IVDs) were dissected from 3 stillborn pups, 3 healthy non-chondrodystrophic (NCD) dogs, and 3 dog patients (Table S1). Complete spines were gently separated from stillborn pups within 24 hours of notice with informed consent from the owner. Complete dog spines (aged 18-24 months) are collected from NCD dogs that had been euthanized in unrelated research studies (approved by the Utrecht University Animal Ethics Committee). We collected and mixed per donor all lumbar and thoracic nucleus pulposus (NPs) (around 20 NPs) from each puppy donor and healthy adult donor, whereafter a single disc sample was used from each patient donor. Redundant surgical material was collected from 3 dog patients scheduled for standard-of-care discectomy surgeries at the veterinary clinics with informed consent from the owner. In all cases, tissues were used for downstream analysis within 24-48 hours after collection and were collected in αMEM (Gibco, #22561) containing 2% bovine serum albumin (BSA; Sigma-Aldrich, #A9418) and 1% Penicillin-Streptomycin (P/S) (Gibco, #15070063). The IVDs from all donors were opened under sterile conditions and the gelatinous NP tissue from the central region was collected by precise separation from the annulus fibrosus (AF)[1]. The areas used in the analysis of NP and AF, are indicated in Fig. 1A.

The NP tissue was collected from patients undergoing dorsal laminectomy and partial microdiscectomy that was performed by an experienced orthopedic surgeon. These patients underwent clinical and orthopedic examination and lumbosacral degenerative disc disease was further evaluated and confirmed on T2-weighted MRI[2]. During surgical microdiscectomy, and following removal of the dorsal annulus fibrosus, the central part of the disc resembling the degenerated nucleus pulposus was collected. For proper referencing, once single-cell datasets were generated, a part of each tissue sample was fixed in formaldehyde and processed for grading of the level of degeneration using a dedicated scoring sheet. Breakdown of parameters used to score degeneration of the tissues, based on Lee et al.[3], is indicated in Table S1-S3. Scoring of degeneration grade was done by an experienced veterinary pathologist (AK) using the online pathology platform Slidescore (https://www.slidescore.com/) using 5 random images for each Hematoxylin and Eosin (H&E), Alcian-blue and Safranin-O / Fast Green stained slide to observe tissue architecture and matrix deposition.

2. Preparation of single-cell suspensions from dog tissue samples

NP and AF tissue single-cell isolation was performed using a modified version of the two-step enzymatic digestion protocol outlined by Williams et al.[1], using a two-step enzymatic digestion, with the enzymes Pronase (Roche, #11459643001) and Collagenase II (Worthington, #443E23628).

NP and AF samples of adult dogs and dog patients were transferred into a sterile $35cm^2$ petri dish and finely minced using a scalpel blade. The tissue fragments were transferred into a 50 mL conical tube containing αMEM+ 2% bovine serum albumin[4]+ 1% penicillin/streptomycin (p/s), and washed at 5.000 rpm, for 5 min to remove any contaminating red blood cells. The residual supernatant was carefully removed and the 50 mL conical tube is filled up to a final volume of 10 mL αMEM + 1% P/S. Optimal digestion was achieved by pooling NPs into one 50 mL conical tube (We collected all lumbar and thoracic NPs (around 20 NPs) into one 50 mL tube for puppy donors, 10 mixed lumbar and thoracic NPs (around 10 NPs) into one tube for healthy adult donors, and 1 NP in one tube for patient donors). Next, 2.5 mL of 0.25% fresh-made Pronase in αMEM + 1% P/S was added to the tube to make a final Pronase concentration of 0.05%. Samples were digested for 30 min, 37°C, on a rotating wheel at 300 rpm. Following digestion, the sample was washed by adding αMEM + 1% p/s until full (50 mL) and centrifuging at 1600 rpm, 5 min, at room temperature (RT). For subsequent digestion with Collagenase type II, the conical tube was filled up to 10mL with αMEM +2% BSA +1% p/s and freshly made, 2.5 mL of 1% collagenase type II creating a final 0.2% collagenase solution. The sample was digested for 2 hours at 37°C, on a rotating wheel at 300 rpm.

For NP tissue samples collected from stillborn puppies, mincing the tissue was not required and the entire tissue was digested in a one-step procedure using 0.03% Collagenase II for 30 min at 37°C while being rotated at 300 rpm. To prepare 0.03% Collagenase II, 0.0015 gram Collagenase was dissolved in 1.0 mL aMEM (0.15% collagenase), whereafter 1 ml of 0.15% collagenase was added to the 4 ml of sample (in aMEM + 2% BSA+ 1% p/s).

Due to the tight junctions between NCs, some cell clumps were expected to remain after enzymatic digestion. Therefore, to further dissociate cell clusters, an additional incubation step with EDTA was performed[1]. The enzymes used in the previous digestion steps were washed in HBSS (Gibco, #14025092) +2% BSA by centrifuging at 1600 rpm for 5 min at RT. The supernatant was removed, and the cells were incubated in 5 mL of 10 mM EDTA, pH8 in PBS for 10 min at 37°C. The cells were washed in HBSS + 2% BSA, to remove any residual EDTA after incubation. For single cell isolation, cells were re-suspended in 2 mL HBSS + 2% BSA and transferred onto a 40 μm cell strainer (Greiner, #542040) to filter for residual cell clusters. Cells that passed through the filter were collected and resuspended in a final volume of 0.5-2 mL HBSS + 2% BSA depending on the starting number of discs. Cells obtained from patient samples and stillborn samples were resuspended in a volume of 200 μL HBSS +2% BSA. The NucleoCounter (NC-200) was used to assess the quality of the single cell isolation by counting the percentage of live cells and diameter of cells and checking any residual amounts of cell clumps and clusters. Well-dissociated, uniform NC populations with a diameter of 11-13 μm and viability exceeding 80% were always observed.

3. Single-cell RNA-seq sample processing

Viable single cells were FACS sorted into 384-well plates, called cell capture plates. Each well of a cell-capture plate contains a small 50 nl droplet of barcoded primers and 10 µl of mineral oil (Sigma M8410). After sorting, plates were immediately spun and placed on dry ice. Plates were stored at -80°C. Single-cell RNA sequencing was performed according to an adapted version of the SORT-seq protocol[5]. Cells were heat-lysed at 65°C followed by cDNA synthesis After second-strand cDNA synthesis, all the barcoded material from one plate was pooled into one library and amplified using in vitro transcription (IVT). Following amplification, library preparation was done following the CEL-Seq2 protocol[6] to prepare a cDNA library for sequencing using TruSeq small RNA primers (Illumina). The DNA library was paired-end sequenced on an Illumina Nextseq™ 500, high output, with a 1×75 bp Illumina kit (read 1: 26 cycles, index read: 6 cycles, read 2: 60 cycles). Single-cell RNA sequencing was performed according to an adapted version of the SORT-seq protocol Muraro et al., 2016[5]. SORT-seq was used in single-objective mode (one cell per drop) with a phase mask, which maintains a low baseline doublet rate of ~4% [7]. This approach was selected to accommodate the large size of vacuolated notochordal cells (~40 µm in diameter), which can exceed the limits of standard microfluidics. SORT-seq also offered flexibility for unpredictable sample availability by allowing plate storage prior to library preparation. Additionally, it provides high sequencing depth and sensitivity[5], improving the detection of lowly expressed genes and the resolution of subpopulations during the NC-to-NPC transition. Single-cell library preparations, Illumina sequencing, were performed by Single Cell Discoveries B.V. (Utrecht, the Netherlands). To minimise batch-associated variation, all plates were processed in parallel through cDNA synthesis, library preparation, and sequencing.

Single-cell RNA seq data analysis:
FASTQ data were processed with the zUMIs pipeline (v 2.9.4f)[8], and the resulting counts table was further analyzed in Rstudio (version 2024.04.2; RStudio Team, 2020) and R (version 4.3.2; R Core Team, 2019) using the Seurat package (v 4.4.0)[9].

FASTQ processing: Reads were demultiplexed, filtered, mapped (STAR v 2.7.3a) and counted (RSubread v2.0.0) using the zUMIs (v2.9.4f) pipeline
Filtering: In order to eliminate low-quality cells and potential doubles, cells with fewer than 1,000 or more than 30,000 reads, and cells with fewer than 500 genes detected, were filtered out. Cells with more than 10% mitochondrial reads were also excluded.
Normalisation: data where normalised and scaled with the SCTransform method v1[10].
PCA and UMAP: Principal component analysis (PCA) was used for dimension reduction using the Seurat function RunPCA() with default parameters. The number of informative principal components was set to 15 based on the flattening decrease of explained variance.
Neighbors were called using FindNeighbors() with default setting and using the first 15 principal components these subsequently clustered with FindClusters() at a resolution of 0.9. Clusters were visualised with Uniform Manifold Approximation and Projection (UMAP) using RunUMAP().

Cluster markers: Differentially expressed genes between clusters were obtained using a parametric Wilcoxon rank sum test at a significance level of $p < 0.05$. Clusters were named based on the a cell signature score calculated with UCell[11] calculated using a pre-determined panels of cell markers for each of the cell types. Differential expression of transcription factors was investigated using the list of transcription factors obtained from the SwissRegulon database[12].

Pseudo-time analysis and single-cell trajectory construction: Pseudo-time analysis was performed with the Slingshot package[13], by using an Omega value of 1.5 to balance the number of edges and their lengths in the minimum spanning tree, ensuring the unbiased reconstruction of differentiation trajectories. Clusters of NC and NP origin were used in the analysis, while AF-derived cells of sclerotome origin were disregarded. Slingshot was then run on UMAP. Pseudo-time for each lineage was split into bins of equal cell numbers. To identifying temporally expressed genes a generalized additive model (GAM) was fitted for each of the 1000 most variable genes using pseudo-time as independent variable.

4. Single cell T-ChIC sample processing and data analysis

To gain an understanding of the interplay between epigenetics and transcription at the level of the individual cell, T-ChIC (Transcriptome + Chromatin ImmunoCleavage) was applied which is a method allowing for the detection of full-length transcripts and histone modifications in the same single cell that we used in maturing disc cells from dog donors. For the down-stream bioinformatic analysis only cells with both a retrieved H3K27me3 profile and transcript were included. T-ChIC combines full-length expression of the total RNA captured using vast transcriptome analysis of single cells by dA-tailing (VASA-seq)[14] and the chromatin state which is assessed with the sort-assisted single-cell chromatin immunocleavage (sortChIC)[15]. For detailed protocol see Blotenburg et.al.[16]. The protocol was modified for small primary cell samples. Three changes were introduced: 1 - Dead cells were stained before cell fixation using Zombie Red, that allows their removal during FACS sorting. This is essential because apoptosis-based DNA fragmentation would otherwise dominate the chromatin experiment. 2 - quantitative reproducible results depend on consistent cell to antibody ratios in all experiments. To ensure stability of conditions independent of sample size, stained K562 carrier cells were added that could be distinguished during FACS sorting. 3 - To avoid loss of sparse primary samples, washing steps are performed using swing bucket centrifugation resulting in a near complete mitigation of cell loss (Fig. S4B-D).

Briefly, this involved:

ProteinA-micrococcal nuclease (PA-MN) Targeting

1. **Cell Preparation**: Spinal disk single cell suspensions were stained with Zombie Red to mark dying cells and washed twice in PBS, before their fixation in precooled 70% EtOH at –20°C for 1h. After fixation cells are washed in once in T-ChIC wash buffer (composed of 47.5 mL RNAse-free water, 1 mL 1 M HEPES pH 7.5, 1.5 mL 5 M NaCl, 3.6 mL spermidine solution, 0.05% Tween-20, EDTA-free protease inhibitor, and 0.2 U/µL RNasin® Plus Ribonuclease

Inhibitor) containing 2mM EDTA, before resuspension in T-ChIC wash buffer containing 1 U/µL RNAsin Plus, 2mM EDTA and 10% DMSO and long term storage at –80C. Thawed cells were centrifuged at 500 g for 4 minutes and washed once with T-ChIC wash buffer containing 2mM EDTA. To maintain a constant antibody to epitope ratio, in these samples where cell yields were low inherent to small tissue samples collected during standard of care surgery, dog cells were mixed with CellTrace Far Red (CTFR) stained human lymphoblast cells (K562 cells, isolated from the bone marrow of a patient with leukaemia).

2. **Antibody Incubation**: Cells were resuspended in 200 µL of T-ChIC wash buffer containing 1 U/µL RNAsin Plus and 2mM EDTA per 0.5 million cells and aliquoted into 0.5 mL protein low binding tubes containing primary H3K27me3 antibody (clone C36B11, 1:100 dilution, NEB, #9733S) diluted in 200 µL T-ChIC wash buffer containing 1 U/µL RNAsin Plus and 2mM EDTA.
3. **Incubation**: Cells were incubated overnight at 4°C on a roller.
4. **Washing**: Cells were washed once with 500 µL T-ChIC wash buffer.
5. **PA-MN Treatment**: Cells were resuspended in 500 µL T-ChIC wash buffer containing 1 U/µL RNAsin Plus, PaMN (3 ng/mL), and Hoechst 34580 (5 mg/mL), and incubated for 1 hour at 4°C on a roller.
6. **Final Washing**: Cells were washed twice with 500 µL T-ChIC wash buffer and passed through a 70 µm cell strainer for sorting.

FACS Sorting

7. **Cell Sorting**: Human K562 cells were removed during FACS based on their CTFR signal and dead dog cells were removed by Zombie Red signal. Single cells were sorted on an Influx machine into 384 well plates for the G1 cell cycle stage based on Hoechst staining. 384-well plates contained 5 µL sterile-filtered mineral oil and 50 nL RNA recovery primer per well.
8. **Sorting Specificity**: Sorting settings included single-objective mode, one drop per sort, complete empty extra coincidence, and a phase mask centred at 10/16.
9. **Data Collection**: Data was collected using BD FACS software version 1.2.0.124.

PA-MN Activation

Volumes for subsequent steps were distributed using a Nanodrop II system (Innovadyme), and plates were spun for 2 minutes at 4°C and 2.000 g after each reagent addition.

10. **Buffer Addition**: 100 nL of Basic T-ChIC buffer without RNAsin Plus, containing 3 mM CaCl2 was added per well to induce Protein A-MN-mediated chromatin digestion.
11. **Incubation**: Plates were incubated for 30 minutes at 4°C in a PCR machine.
12. **Reaction Stopping**: The reaction was stopped by adding 100 nL stop solution (containing 40 mM EGTA, 1.5% NP40, 2 mg/mL temperature labile proteinase K, and 12.5 mM MgCl2) and incubated for 20 minutes at 4°C.

13. **Chromatin Release**: Plates were incubated at 37°C for 2 hours for chromatin release and cell lysis, followed by heat inactivation at 55°C for 20 minutes. Plates were stored at -80°C until further processing.

Library Preparation

14. **RNA Fragmentation**: Thawed RNA was fragmented at 85°C for exactly 2 minutes and then cooled on ice.
15. **RNA Repair and Poly-A Tailing**: RNA fragments were repaired and poly-A-tailed by adding 150 nL of RNA repair mix per well and incubating at 37°C for 1 hour. The RNA repair mix per plate included 18.75 µL nuclease-free water, 12 µL 0.1 M DTT, 0.9 µL 1 M MgCl2, 18 µL 1 M KCl, 16.8 µL 1 M Tris (pH 8), 4.5 µL 50% PEG8000, 0.45 µL 20 mg/mL BSA, 5.25 µL 0.1 mM ATP, 6 µL RNase-OUT, 1.05 µL polyA polymerase, and 7.20 µL T4 PNK.
16. **Reverse Transcription**: Repaired RNA fragments were reverse transcribed by adding 100 nL of RT mix per well and incubating at 50°C for 1 hour. The RT mix per plate contained 38.7 µL nuclease-free water, 3 µL 0.1 M DTT, 4.5 µL 1 M KCl, 3 µL 50% PEG8000, 0.3 µL 20 mg/mL BSA, 4.5 µL 10 mM dNTPs, and 6 µL SuperScript III.
17. **Blunt-Ending DNA Fragments**: DNA fragments were blunt-ended by adding 150 nL DNA repair mix per well, incubating for 30 minutes at 37°C, followed by 20 minutes at 75°C for enzyme inactivation. The DNA repair mix per plate consisted of 54.6 µL nuclease-free water, 1.2 µL 5 M NaCl, 10.5 µL 1 M Tris (pH 7.5), 1.5 µL 1 M MgCl2, 4.5 µL 0.1 M DTT, 4.5 µL 50% PEG8000, 0.45 µL 20 mg/mL BSA, 3.9 µL 100 mM ATP, 1.95 µL 10 mM dNTPs, 3 µL TL Exo1, 1.95 µL Klenow large fragment, and 1.95 µL T4 PNK.
18. **A-Tailing**: Blunt fragments were A-tailed by adding 150 nL A-tailing mix per well and incubating for 15 minutes at 72°C. The A-tailing mix per plate included 76.7 µL nuclease-free water, 6 µL 1 M KCl, 4.5 µL 50% PEG8000, 0.45 µL 20 mg/mL BSA, 1.2 µL 100 mM dATP, and 1.2 µL Taq360.
19. **Adapter Ligation**: DNA adapters containing a unique molecular identifier [17]. For adapter ligation, 50 nL of 5 uM adapters in 50 mM Tris (pH 7) was added to each well with the Mosquito HTS (TTP Labtech).

Centrifugation and Ligation Mix Addition: After centrifugation, 150 nL of adapter ligation mix was added and plates were incubated for 20 minutes at 4°C, followed by 16 hours at 16°C for ligation and 10 minutes at 65°C for ligase inactivation. The adapter ligation mix per plate included 17.1 µL nuclease-free water, 2.1 µL 1 M MgCl2, 2.7 µL 1 M Tris (pH 7.5), 40.5 µL 0.1 M DTT, 4.5 µL 50% PEG8000, 0.45 µL 20 mg/mL BSA, 2.1 µL 100 mM ATP, and 21 µL 400,000 units/mL T4 DNA Ligase.

20. **Minimizing Material Loss**: Before pooling, 1 uL of nuclease-free water was added to each well.
21. **Pooling Ligation Products**: Ligation products were pooled by centrifugation into oil-coated VBLOK200 reservoirs (ClickBio) at 500 g for 2 minutes.
22. **Liquid Phase Transfer**: The liquid phase was transferred into 1.5 mL Eppendorf tubes.

23. **Purification**: The liquid phase was purified by centrifugation at 13,000 g for 1 minute and transferred into a fresh tube. Steps 23 and 24 were repeated twice.
24. **DNA Fragment Purification**: DNA fragments were purified using Ampure XP beads (Beckman Coulter) prediluted 1:8 in bead binding buffer (1 M NaCl, 20% PEG8000, 20 mM Tris pH 8, 1 mM EDTA) at a bead-to-sample ratio of 0.8.
25. **Bead Incubation and Washing**: After 15 minutes of incubation at room temperature, beads were washed twice with 1 mL 80% ethanol, resuspending the beads during the first wash.
26. **Resuspension**: Beads were resuspended in 17 µL nuclease-free water, eluted for 2 minutes, and transferred into a fresh 0.5 mL tube.
27. **Second-Strand Synthesis**: Second-strand synthesis was performed using the NEBNext® Ultra™ II Non-Directional RNA Second Strand Synthesis Module. Per plate, 2 µL 10x reaction buffer was mixed with the sample on ice, followed by the addition of 1 µL enzyme, gentle mixing, incubation at 16°C for 2.5 hours, and heat inactivation at 65°C for 20 minutes.
28. **Second Cleanup**: A second cleanup was performed by adding 26 µL of undiluted Ampure XP beads, resuspending the beads in 8 µL nuclease-free water.
29. **In Vitro Transcription**: DNA was linearly amplified by in vitro transcription using 12 µL of the MEGAscript™ T7 Transcription Kit (Fisher Scientific) for 12 hours at 37°C.
30. **RNA Purification**: The produced RNA was purified using RNA Clean XP beads (Beckman Coulter) with a 1.3 bead-to-sample ratio (26 µL beads), followed by sample resuspension in 12 µL nuclease-free water.
31. **Storage and rRNA Depletion**: At this stage, 6 µL aRNA could be stored at -80°C, while the remaining 6 µL continued to rRNA depletion using 2 µL hybridisation buffer (1000 mM NaCl in 500 mM Tris pH 7.5) and 2 µL 25 µM rRNA depletion oligos.
32. **Oligo Hybridisation**: Oligos were hybridised to their rRNA targets by incubation at 95°C for 2 minutes, followed by a gradual temperature decrease of 0.1°C/s down to 45°C. Once at 45°C, 10 µL prewarmed RNAse mix (2 µL thermostable RNAseH (Epicentre), 8 µL RNAse buffer: 250 mM NaCl, 50 mM $MgCl_2$ in 125 mM Tris pH 7.5) was added and samples were incubated at 45°C for another 30 minutes.
33. **DNA Template and Probe Removal**: DNA template and unused depletion probes were removed by adding 4 µL RNase-free DNAse (Promega) with 5 µL 10 mM $CaCl_2$ in 30 µL nuclease-free water, followed by incubation at 37°C for 30 minutes.
34. **RNA Purification**: RNA was purified using 80 µL RNA Clean XP beads per sample and eluted in 6 µL water.
35. **Adapter Ligation for RNA**: 5 µL of the supernatant was transferred to a new 0.5 mL Eppendorf tube for RNA adapter ligation. For this, 1 µL 20 µM RA3 (/5rApp/TGGAATTCTCGGGTGCCAAGG/3SpC3/) was added, mixed and incubated for 2 minutes at 70°C, followed by cooling on ice. On ice, 4 µL RNA ligation mix was added, mixed by pipetting, and incubated for 1 hour at 25°C (lid temperature should not exceed 25°C). The RNA ligation mix per sample contained 1 µL T4 RNA ligase 2, truncated (NEB), 1 µL T4

RNA ligase reaction buffer (NEB), 1 μL RNAseOUT (Invitrogen), and 1 μL nuclease-free water.

36. **Priming for Reverse Transcription**: RNA was primed for reverse transcription by adding 1 μL of 10 mM dNTPs and 2 μL of 20 mM reverse transcription primer (RTP: GCCTTGGCACCCGAGAATTCCA).
37. **Hybridization**: The mixture was incubated at 65°C for 5 minutes, followed by immediate cooling on ice.
38. **Reverse Transcription**: Reverse transcription was initiated by adding 4 μL of first strand buffer (Invitrogen), 1 μL of 0.1 M DTT, 1 μL of RNaseOUT (Invitrogen), 1 μL of SuperscriptIII (Invitrogen), and 1 μL of nuclease-free water. The mixture was then incubated at 50°C for 1 hour, followed by 15 minutes at 70°C.
39. **Purification of Single-Stranded DNA**: Single-stranded DNA was purified using 1 μL of RNAseA (Thermo Fisher) and incubated for 30 minutes at 37°C.
40. **DNA Cleanup**: The DNA was cleaned up with 22 μL of Ampure XP beads and resuspended in 20 μL of nuclease-free water, with 10 μL stored at -80°C.
41. **Library PCR for Barcode Addition**: The library PCR to add Illumina small RNA barcodes and handles was performed by adding 25 μL of NEBNext Ultra II Q5 Master Mix (NEB, M0492L), 11 μL of nuclease-free water, and 2 μL each of RP1 and RPIx primers (10 mM, see Illumina small RNA indexing primer sequences) to the 10 μL single-stranded DNA product.
42. **PCR Incubation**: The PCR incubation included activation for 30 seconds at 98°C, followed by 8 cycles of 10 seconds at 98°C, 30 seconds at 60°C, and 30 seconds at 72°C, with final amplification for 10 minutes at 72°C.
43. **PCR Product Cleanup**: PCR products were cleaned using two consecutive DNA bead clean-ups with a 0.8X bead-to-sample ratio, eluting in 30 μL of nuclease-free water after the first round and in 11 μL for the final product.
44. **Quality Assessment**: Abundance was determined using a dsDNA HS QUBIT kit, and after adjusting concentrations to approximately 2 ng/μL, fragment size distribution was assessed using a DNA HS bioanalyzer.

PA-MN Production

The PA-MN fusion protein was produced according to the methods section in Schmid et al[18].

Sequencing

Final DNA libraries were sequenced using paired-end 100 bp reads on a FrNextSeq2000.

Transcriptome Analysis

Fastq files were processed into .loom files using the T-ChIC Snakemake (see https://github.com/marloes3105/tchic/tree/main/workflows/1.snakemake-workflow/, v1.0). The pipeline includes the following steps:

1. **Demultiplexing**: Reads were demultiplexed for CelSeq2 barcodes using SingleCellMultiOmics' (SCMO) demux.py (version 0.1.30).
2. **Trimming**: PolyA tails were trimmed using SCMO trim_vasa.py and adapters were trimmed using cutadapt (version 4.1).
3. **Mapping**: Reads were mapped to the canine (CanFam3.1) genome (Ensembl 104) using STAR (version 2.5.3a).
4. **Tagging and Deduplication**: Reads were tagged and deduplicated using SCMO bamtagmultiome.py.
5. **Generating .loom Files**: Velocyto (version 0.17.17) was used to generate .loom files.
6. **Filtering**: Cells with fewer than 1,000 or more than 50,000 reads, and cells with fewer than 100 genes detected, were filtered out. Cells with more than 0.6% mitochondrial reads were also excluded.
7. **Normalization and Exclusion**: Mitochondrial reads, Malat1, SnoRNAs, and ribosomal reads were excluded. Counts were normalised to 1,000 transcripts per cell and logarithmized. The number of counts and percentage of mitochondrial reads were regressed out. Each gene was scaled to unit variance with a maximum of 10.
8. **PCA and UMAP**: PCA was performed to filter out cells with low ChIC quality. Batches were integrated using harmony. Neighbors were calculated using sc.pp.neighbors (settings: n_neighbors = 20, n_pcs = 30). UMAP was generated using sc.tl.umap (settings: min_dist = 0.5, spread = 1.0).
9. **Clustering**: Cells were clustered using scanpy leiden clustering at resolution 0.9.
10. **Differential expression**: Differential gene expression was calculated with rank_genes_groups(method = 'wilcoxon') using a Wlcoxon rank sum test. Cell types were assigned to each cluster based on marker gene expression.
11. **RNA velocity**: RNA velocities are obtained by modelling RNA splicing kinetics of actively transcribed (unspliced) and mature (spliced) mRNA counts for each gene with positive velocity indicating that a gene is up-regulated (more unspliced mRNA) and negative velocity (more spliced mRNA) indicating gene downregulation. The combination of velocities across genes is then used to estimate the future state of an individual cell. RNA velocity was calculated with scVelo (version 0.3.2) [19]. Using a dynamical model to learn the transcriptional dynamics with scv.tl.recover_dynamics() and scv.tl.velocity(mode="dynamical"). Pseudotime was calculated with scv.tl.velocity_pseudotime().

ChIC Analysis

Fastq files were processed into count tables using the T-ChIC Snakemake (see https://github.com/marloes3105/tchic/tree/main/workflows/1.snakemake-workflow/, v1.0). The pipeline includes the following steps:

1. **Demultiplexing**: Reads were demultiplexed for T-ChIC barcodes using SingleCellMultiOmics' (SCMO) demux.py (version 0.1.30), with a hamming distance of 0.
2. **Trimming**: Adapters were trimmed using cutadapt (version 4.1).

3. **Mapping**: Reads were mapped paired-end to the canine (CanFam3.1) genome (Ensembl 104) using bwa mem (version 0.7.16a) with parameters -M -I 1000.
4. **Tagging and Filtering**: Reads were tagged with SCMO bamtagmultiome.py. Duplicates and reads with an insert size above 1000, mapping quality below 50, or known-TA ligation motif were removed.
5. **Quality Control**: To exclude low-quality cells, transcript-based PCs were used to determine neighbourhoods of comparable cells. The local chromatin enrichment of each cell was compared to the neighbourhood average. Pseudobulks of total count normalised cells were created to determine the highest covered bins containing 80% of the reads. The read fraction of the cell of interest in these bins was determined, and cells with an enrichment of 2 standard deviations below average were marked as low quality.
6. **Cell selection Script**: The script cellSelection.py was used with the following parameters: -neighbours number of neighbours to use, -cutoff minimal number of reads, -chic_csv genome-wide ChIC count table/merged_count_table_50000.csv, -trans_csv UMAP exported from Scanpy, -o folder name for output. For all datasets, 50 neighbours were used, and the minimum reads per cell were set at 500 for H3K27me3. Results in good and bad cells based on enriched bin coverage in neighbour cells.
7. G**enerate TSS counts: Using the** bamCountRegions.py script a count table with H3K27Me3 counts in a region 5kb around the TSS of each gene was made. Using the BAM file of the "good" cells and a BED file for the 10kb TSS regions.
8. **Scanpy analysis:** To visualise the H3K27Me3 signal the H3K27Me3 count table was loaded into a new layer (k27_TSS) of the scanpy transcriptome adata object. Subsequently the H3K27Me3 counts were quantile normalised. PCA was performed with sc.tl.pca() and batches were integrated using Harmony, sce.pp.harmony_integrate(). Neighbours were calculated using sc.pp.neighbors (settings: n_neighbors = 20, n_pcs = 30) UMAP was generated using sc.tl.umap (settings: min_dist = 0.5, spread = 1.0). Clustering the cells using the Leiden method using a resolution of 0.9 with sc.tl.leiden(resolution=0.9) Differentially methylated genes were calculated for differentially expressed genes using sc.tl.rank_genes_groups(method='wilcoxon', layer='k27_TSS_quant', use_raw=False), using a Wilcoxon ranks sum test.

5. Human NPCs study

Human NP tissue collection

Human tissues were resected from five adult human body donors (Table S4). Bodies were donated through a body donation program to the Anatomy Department of the University Medical Center Utrecht (UMCU), the Netherlands. Informed consent was obtained during life, allowing the use of these bodies for educational and research purposes. Tissues were collected within 48 hours after death. In a sterile biosafety cabinet, the spine was disinfected in betadine for 5 minutes, rinsed, and dried. Excess fat and soft tissues were removed using sterile scalpels and forceps. The tissue was submerged in 70% ethanol for 5 minutes, dried, and dissected.

Primary cell culture of human NPCs

NP tissue collected from human discs were washed twice with DMEM (Gibco, #31966-021) adjusted to 400 mOsm with 2.2 mg/mL NaCl and supplemented with antibiotics (2% penicillin/streptomycin, 2.5 μg/mL Amphotericin B, 50 μg/mL Gentamicin, all from Gibco), and minced into ~2x2x2 mm pieces. The collected NP tissue was enzymatically digested for 1 hour with 0.2% w/v Pronase (Roche, #11459643001) in DMEM (Gibco) with antibiotics, followed by 12–14 hours at 37°C in 0.05% w/v Collagenase type II (Worthington) in DMEM/antibiotics. The cell suspension was obtained by filtering through a 70 μm cell strainer. Cell pellets were resuspended in expansion medium P0 (DMEM + 10% FBS + antibiotics) and seeded at 5,000 cells/cm² in culture flasks. Upon cell attachment, bFGF (R&D, 1 ng/mL) was added. Cells reached 80% confluence after 5-8 days and were cryopreserved at passage 1.

Bulk T-ChIC of human NPCs

Cryopreserved P1 cells were used directly for the TCHiC as detailed in section 4 using the protocol described in Blotenburg et al.[16] Note that the FACS sorting step is omitted in the bulk sequencing protocol. Final DNA libraries were sequenced using paired-end 100 bp reads on a NextSeq2000. For transcript and ChIC analysis, Fastq files were processed into count tables using the T-ChIC Snakemake (see https://github.com/marloes3105/tchic/tree/main/workflows/1.snakemake-workflow/, v1.0). Following demultiplexing, for the transcript, reads were mapped to the human (hg38) genome using STAR (version 2.5.3a). For ChIC, reads were mapped paired-end to the human (hg38) genome using bwa mem (version 0.7.16a) with parameters -M -I 1000.

Gene specific H3K27me3 enrichment was quantified using the bamCountRegions.py script, which generated a count table based on reads within 10 kb window around the transcription start sites[20] of annotated genes. Gene-level RNA counts and TSS-level H3K27me3 counts were aggregated per sample and normalised using median ratio normalisation for downstream visualisation (Data file S4 and S5). For track plots, 1 kb-resolution bigWig files were generated using bamCoverage (Samtools).

H3K27me3 removal and CRISPRa mediated activation of TBXT in human NPCs

The H3K27me3-based repression is deposited by the Polycomb repressive complex 2 (PRC2), catalyzing the mono-, di-, and trimethylation of histone H3 lysine 27[21]. To check the effect of H3K27me3 removal in the degenerate human NPCs, 1 million P1 cryopreserved human NPCs from degenerated discs were equally seeded in two T75 culture flasks. One flask was cultured with expansion medium (hgDMEM; 2.2 mg/ml NaCl (Sigma-Aldrich, #31434-1KG-M); 10% FBS (Biowest, #S1810-500); 1% p/s) and the other was cultured in expansion medium with 500 nM GSK126 (EZH2 inhibitor, Sigma Aldrich). The 500 nM concentration of GSK126 has been widely used in previous studies and showed a pronounced global reduction of H3K27me3[22-24]. After 1-week expansion at 37 ºC, 5% CO2 and 20% O2 levels, NPCs were dissociated and seeded in expansion medium in 24-well plates (20,000 cells /cm^2). After five days, the CRISPR activation

system (CRISPRa), dCas9-SAM was used to activate *TBXT* transcription using a specific gRNA targeting vector designed to target the promotor region of the *TBXT* gene using p2T-gRNA (template from addgene, #71485) vector backbone. The gRNA targeting vector was co-transfected with PB-SAM-Dest, a SAM vector (addgene, #102563) to achieve transcriptional activation at the *TBXT* promoter[25]. The dCas9–SAM transactivation system recruits transcriptional activators to gene promoters and facilitates local chromatin remodelling[26]. The CRISPRa plasmids transfection was carried out within 24 hours using Lipofectamine3000 (Invitrogen) following the commercial protocol. The transfected cells were collected for RT-qPCR 48 hours after transfection.

Two housekeeping genes (*GAPDH* and *RPS5*) were included, and their expression remained stable across all treatment groups, which confirms that the lipofectamine treatment did not induce a global or non-specific transcriptional shift in the human NPCs.

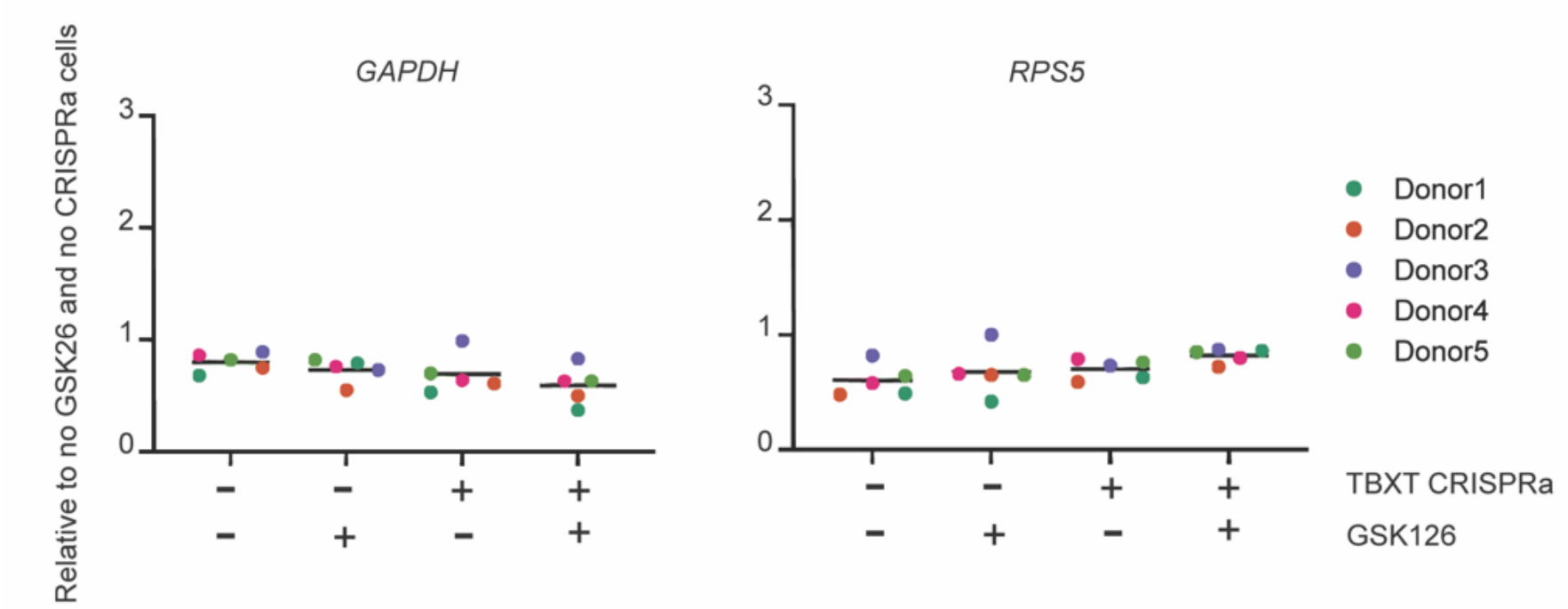

The relative expression of *GAPDH* and *RPS5* genes following GSK126 treatment and combined GSK126 + CRISPRa activation.

6. Reverse transcription quantitative PCR

To examine the mRNA expression of pluripotency markers, mesendoderm markers, and notochordal markers, the total RNA from monolayer cells was extracted using RNeasy mini kit (Qiagen, #74106) and RNA from pellets was extracted using RNeasy micro kit (Qiagen, #74004). The cDNA was then synthesized using Iscript cDNA synthesis kit (Bio-Rad, #1708891) for quantitative reverse transcription PCR detection. Relative quantification was calculated using the efficiency-corrected $\Delta\Delta C_T$ method as described below.

For each target gene, the C_T-value was normalised to the geometric mean C_T-value of the reference genes (*GAPDH* and *RPS5*) using the formula: ΔC_T target=C_T target – C_T geomean ref. The geometric mean of the reference genes was used, because expression levels behave multiplicatively, not additively. This ΔC_T value was employed in the statistical analysis.

For the set of experiments shown in Figures 6E and S6F), the mean ΔC_T-value of all samples was used as calibrator in the following formula: $\Delta\Delta C_T$=ΔC_T target – ΔC_T mean all samples. Thereafter, the E-$\Delta\Delta C_T$-value was calculated to determine fold change of induction. In this formula, E indicates the amplification efficiency (between 95% and 105%) of the target gene. The primer sequences are listed in Table S7 below.

7. Histopathological evaluation

For histological analysis, the IVD sample was fixed in 4% paraformaldehyde for no longer than seven days. The fixed samples were embedded in paraffin and sectioned them into 5mm-thick slices. The hematoxylin and eosin (H&E), Safranin-O/Fast Green staining and Alcian-blue/Picrosirius red staining were performed on the IVD sections following previously established protocols[27]. Scoring was conducted by an experienced veterinary pathologist using Table S1-S3 parameters for grading. The online pathology platform Slidescore (https://www.slidescore.com/) was used for grading within 5 random images for each H&E, Alcian-blue/Picrosirius red and Safranin-O/Fast Green-stained sections to observe cell morphology, tissue architecture, and matrix deposition.

Immunohistochemical staining was performed to evaluate the expression of IBA1 in AF tissue, and Brachyury (TBXT), SOX9, and collagen type I (COL1) in all formalin-fixed paraffin-embedded tissue samples. Tissue sections were deparaffinized in xylene and rehydrated through a graded ethanol series to distilled water. Antigen retrieval was performed according to antigen-specific protocols (see Table S8). Endogenous peroxidase activity was blocked by incubation with hydrogen peroxide, followed by a blocking step to reduce non-specific binding. Sections were incubated overnight at 4°C with primary antibodies against IBA1, TBXT, SOX9, and COL1 at optimized dilutions (see Table S8). Immunoreactivity was visualized using 3,3′-diaminobenzidine (DAB)[28] as the chromogen, resulting in a brown precipitate. Sections were counterstained with hematoxylin, dehydrated through graded ethanol, cleared in xylene, and cover slipped. Staining specificity was assessed using isotype-matched control antibodies in place of the primary antibody. Detailed information on antibody sources, dilutions, antigen retrieval conditions, and incubation parameters is provided in Table S8.

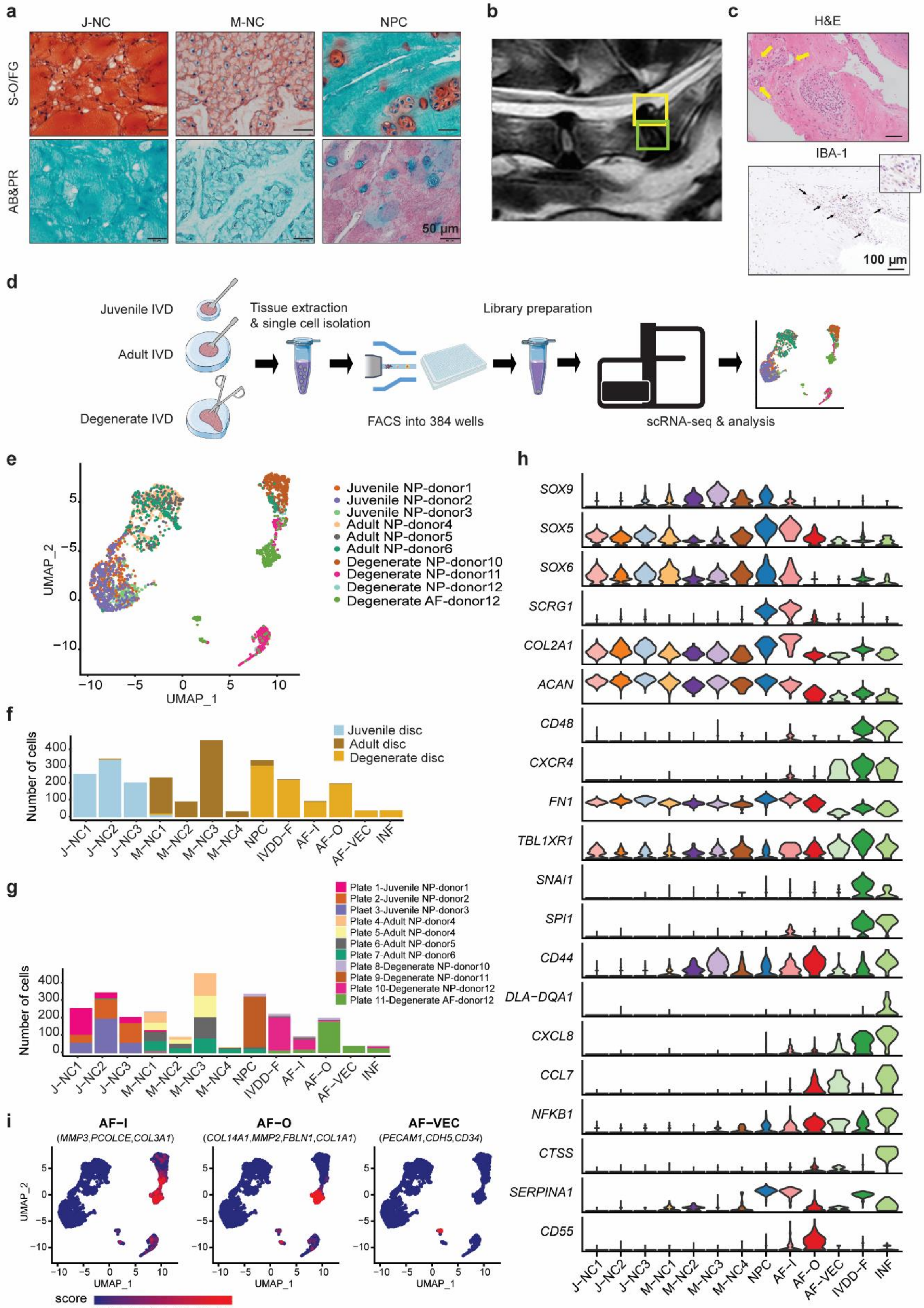
a
J-NC
M-NC
NPC
S-O/FG
AB&PR
50 µm
b
c
H&E
IBA-1
100 µm
d
Juvenile IVD
Adult IVD
Degenerate IVD
Tissue extraction
& single cell isolation
FACS into 384 wells
Library preparation
scRNA-seq & analysis
e
UMAP_2
UMAP_1
Juvenile NP-donor1
Juvenile NP-donor2
Juvenile NP-donor3
Adult NP-donor4
Adult NP-donor5
Adult NP-donor6
Degenerate NP-donor10
Degenerate NP-donor11
Degenerate NP-donor12
Degenerate AF-donor12
f
Number of cells
Juvenile disc
Adult disc
Degenerate disc
J-NC1 J-NC2 J-NC3 M-NC1 M-NC2 M-NC3 M-NC4 NPC IVDD-F AF-I AF-O AF-VEC INF
g
Plate 1-Juvenile NP-donor1
Plate 2-Juvenile NP-donor2
Plaet 3-Juvenile NP-donor3
Plate 4-Adult NP-donor4
Plate 5-Adult NP-donor4
Plate 6-Adult NP-donor5
Plate 7-Adult NP-donor6
Plate 8-Degenerate NP-donor10
Plate 9-Degenerate NP-donor11
Plate 10-Degenerate NP-donor12
Plate 11-Degenerate AF-donor12
Number of cells
J-NC1 J-NC2 J-NC3 M-NC1 M-NC2 M-NC3 M-NC4 NPC IVDD-F AF-I AF-O AF-VEC INF
h
SOX9
SOX5
SOX6
SCRG1
COL2A1
ACAN
CD48
CXCR4
FN1
TBL1XR1
SNAI1
SPI1
CD44
DLA-DQA1
CXCL8
CCL7
NFKB1
CTSS
SERPINA1
CD55
J-NC1 J-NC2 J-NC3 M-NC1 M-NC2 M-NC3 M-NC4 NPC AF-I AF-O AF-VEC IVDD-F INF
i
AF-I
(MMP3,PCOLCE,COL3A1)
AF-O
(COL14A1,MMP2,FBLN1,COL1A1)
AF-VEC
(PECAM1,CDH5,CD34)
UMAP_2
UMAP_1
score
0.3 0.4 0.5 0.6 0.7 0.8 0.9

Fig. S1 Defining the major IVD cell types in NCD dogs. Three individual samples per life stage were utilized for the juvenile, healthy and degenerate adult NP. **a** Safranin-O/Fast green (S-O/FG) staining and Alcian blue and picrosirius red staining (AB&PR) of the NP tissue show matrix composition, with the ECM becoming depleted from the glycosaminoglycan-rich matrix in degeneration. **b** On T2-weighted sagittal MRI, the L6-7 spinal segment presents with a normal IVD with a homogenous structure and a hyperintense signal, indicating high water content and normal IVD height (Pfirrmann grade 2; Donor 10 in Table S1). The degenerative lumbosacral disc is hypointense (green box), lacks distinction between the NP and AF, and presents with moderate collapse (Pfirrmann grade 4). The yellow box illustrates a hyperintensity zone within the dorsal AF. **c** H&E and immunohistochemistry stained tissue sections from the harvested AF tissue of this patient showing extensive neovascularization (yellow arrows) adjacent to an area of mononuclear cell infiltration. IBA-1 immunopositivity confirms that the majority of these cells are positive (black arrows) indicating a histiocytic origin, consistent with findings previously reported for tissues presenting with a hyperintensity zone within the dorsal AF [29]. This was the single patient dog AF tissue used in the single-cell transcriptomic study. **d** The SORT-seq experimental workflow. **e** Transcriptome-based UMAP projection (2,597 cells; 10,891 UMI/cell; 3,533 genes/cell) of cell populations annotated by sample origin. **f-g** Contribution of donor source and sequencing run to each cluster. **h** Violin plot showing the expression of cluster specific genes with normalised expression levels. The NC and NPC cluster-specific genes are highlighted in Fig. 1f. **i** Identification of clusters (AF-I, AF-O, AF-VEC) using the average combined marker score using UCell. IVD, intervertebral disc; FACS, fluorescence associated cell sorting; SC-RNA, single-cell RNA sequencing; NC, notochordal cells including both J-NC and M-NC clusters; J-NC, juvenile NC; M-NC, mature NC; NPC, nucleus pulposus cell; IVDD-F, IVD degeneration associated fibrocyte; AF, annulus fibrosus; AF-I, inner AF; AF-O, outer AF; AF-VEC, AF-vascular endothelial cell; INF, inflammatory cell.

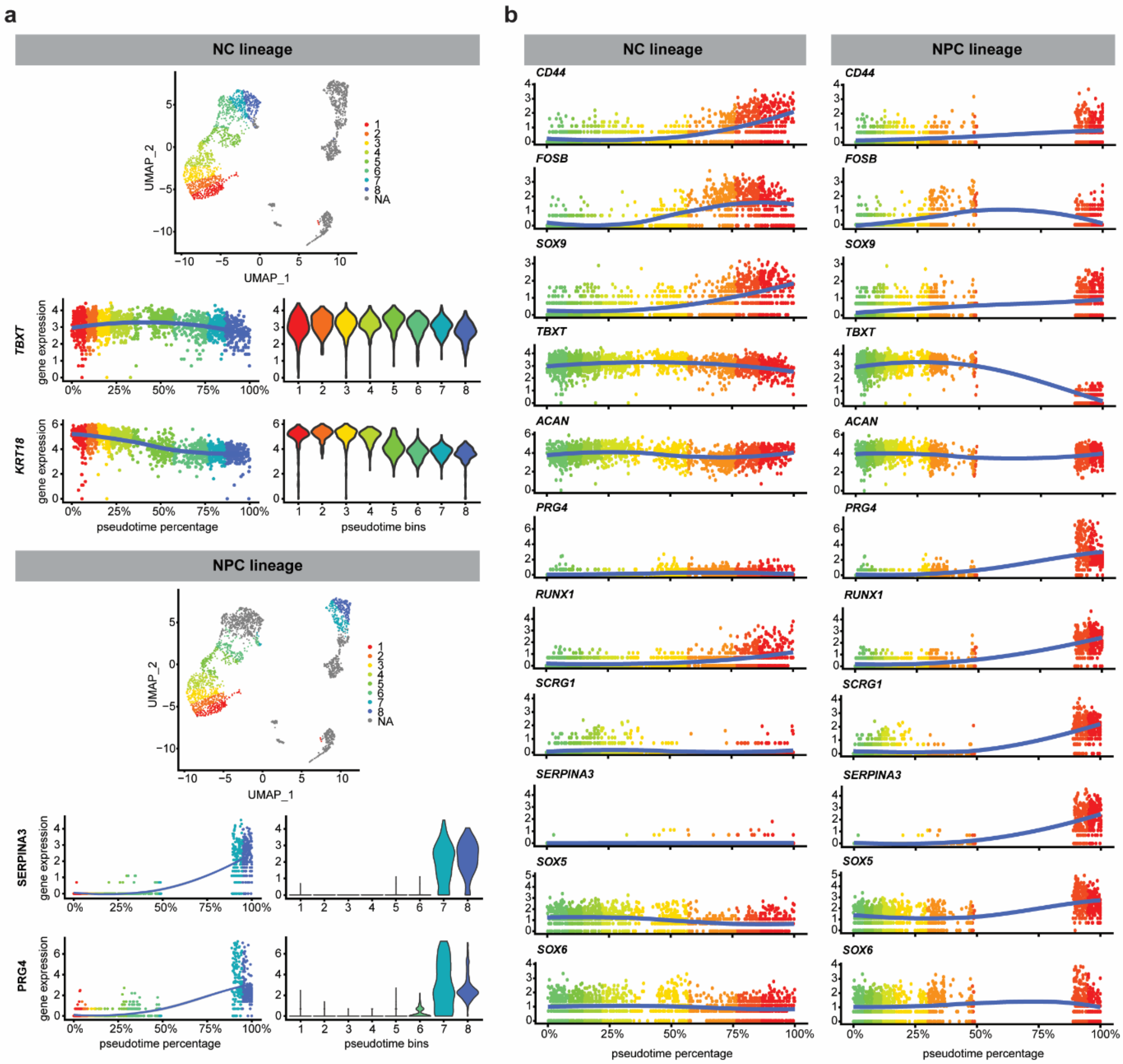

Fig. S2 Exploring NC hierarchies across IVD ageing and degeneration. **a** UMAP of cells grouped into 8 pseudotime bins for each lineage. For each lineage, for the individual cells within each pseudotime bin fitted gene expression changes are shown (left) and corresponding Violin plots of normalised gene expression on the right. They are ordered along pseudotime and coloured according to the pseudotime bins. Two genes are selected to represent each lineage (*TBXT*, *KRT18* for NC lineage and *SERPINA3*, *PRG4* for NPC lineage). **b** Fitted gene expression changes for individual cells comparing NC and NPC lineages.

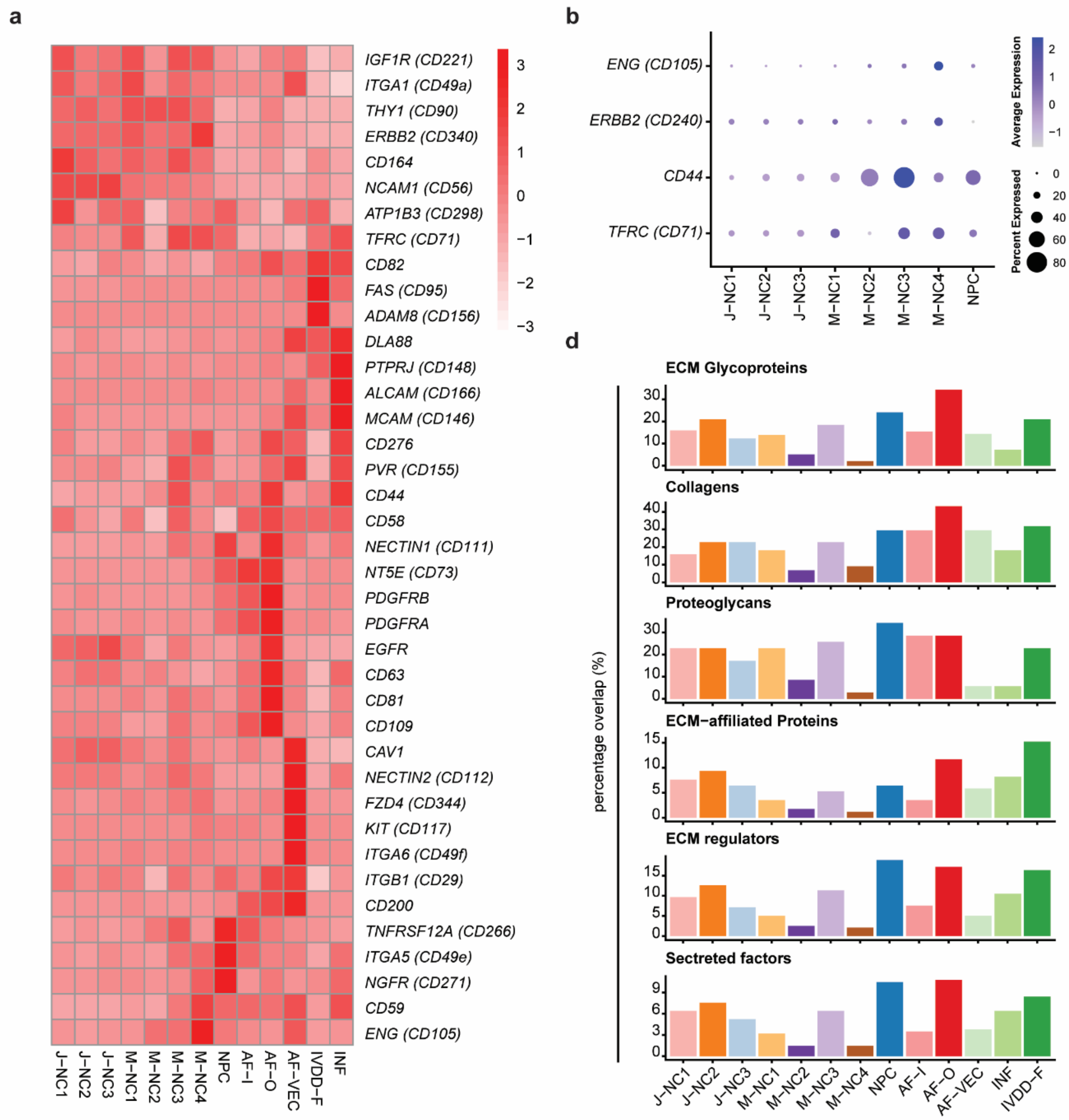

c

J-NC1	J-NC2	J-NC3	M-NC1	M-NC2	M-NC3	M-NC4	NPC	IVDD-F
MZF1	*TBXT*	*TBXT*	***ZBTB16***	***FOSB***	***FOSB***	***RFX3***	***GLIS3***	*SPI1*
ELF3	***CUX2***	***BHLHE40***	*ID4*	***JUND***	*NFATC2*	*NFATC2*	*SOX5*	*TBL1XR1*
NR3C2	***MAX***	*GLIS1*	*TBXT*	*NR4A1*	***JUN***	***FOSB***	*RUNX1*	*NR4A3*
LMX1A	***BHLHE40***	***CUX2***	***LMX1A***	***FOXJ1***	***RFX3***	***POU2F2***	*TCF7L2*	*SNAI1*
CUX2	***TFCP2***	*NR3C2*	***BARX2***	*HIF1A*	*SOX9*	***SOX13***	*KLF3*	*KLF6*
BHLHE40	*GLIS*	***MITF***	*HIF1A*	*ATF4*	***JUND***	*NR4A1*	*ELK3*	*CREM*

Fig. S3 Exploring NC phenotypes across cellular hierarchy. **a** Heatmap showing representative surface markers expression in each cell cluster. To provide insights into potential methods for identifying and isolating these cells, we analyzed different cell surface markers[30,31] but did not identify unique discriminating cell surface markers. **b** Scaled dot plot showing the mean normalised expression of key cell surface markers for each cluster with dot size indicating the percentage of cells in each cluster with detected expression. *TFRC* (*CD71*) was highly expressed in around 40% of both M-NC3 and M-NC4 populations, with over 80% of the M-NC3 population showing *CD44* expression, in contrast to approximately 30% of the M-NC4 population expressing high levels of the tyrosine kinase receptor, *ERBB2* (*CD240*) and *ENG* (*CD105*) an accessory receptor for TGF-β[32]. From a translational perspective, this suggests that there are likely no unique discriminating cell surface markers for effective differential cell sorting. **c** The top six differentially expressed transcription factors (TFs) of each cell cluster compared to all other cell clusters which have been associated with the human or mammalian IVD and several only with the zebrafish or Ciona intestinalis notochord which are marked in bold[33-39]. TFs not previously associated with the IVD are marked in bold red. Differential TF expression was investigated using the SwissRegulon database. **d** The percentages of DEGs associated with six matrisome patterns (collagens, glycoproteins, proteoglycans, ECM regulators, ECM affiliated, and secreted factors in each cluster. The matrisome patterns are classified according to the matrisome classification database[40].

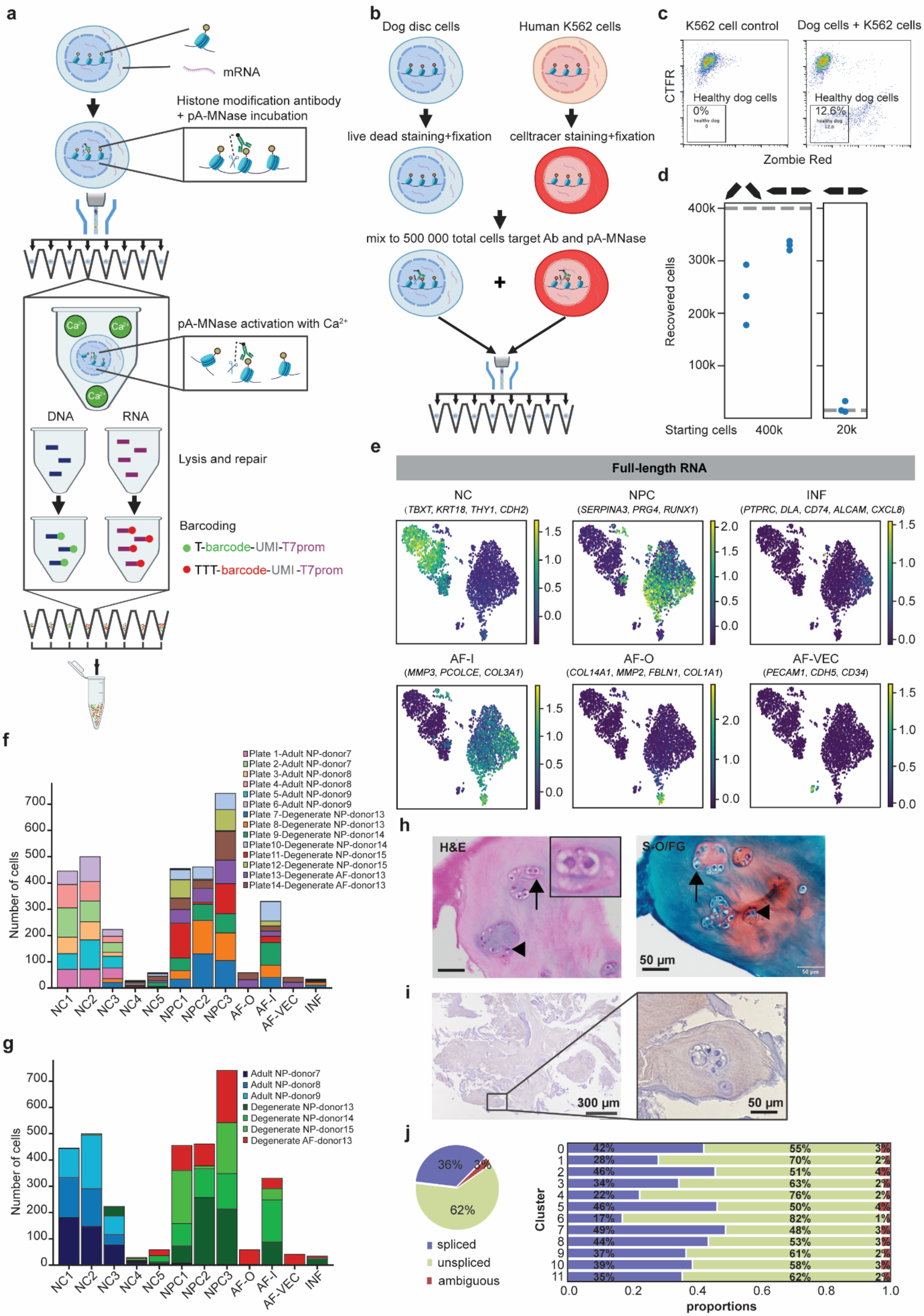

a
mRNA
Histone modification antibody + pA-MNase incubation
pA-MNase activation with Ca2+
DNA
RNA
Lysis and repair
Barcoding
T-barcode-UMI-T7prom
TTT-barcode-UMI-T7prom
b
Dog disc cells
Human K562 cells
live dead staining+fixation
celltracer staining+fixation
mix to 500 000 total cells target Ab and pA-MNase
c
K562 cell control
Dog cells + K562 cells
CTFR
Healthy dog cells
0%
12.6%
Zombie Red
d
Recovered cells
Starting cells
400k
20k
e
Full-length RNA
NC (TBXT, KRT18, THY1, CDH2)
NPC (SERPINA3, PRG4, RUNX1)
INF (PTPRC, DLA, CD74, ALCAM, CXCL8)
AF-I (MMP3, PCOLCE, COL3A1)
AF-O (COL14A1, MMP2, FBLN1, COL1A1)
AF-VEC (PECAM1, CDH5, CD34)
f
Number of cells
Plate 1-Adult NP-donor7
Plate 2-Adult NP-donor7
Plate 3-Adult NP-donor8
Plate 4-Adult NP-donor8
Plate 5-Adult NP-donor9
Plate 6-Adult NP-donor9
Plate 7-Degenerate NP-donor13
Plate 8-Degenerate NP-donor13
Plate 9-Degenerate NP-donor14
Plate10-Degenerate NP-donor14
Plate11-Degenerate NP-donor15
Plate12-Degenerate NP-donor15
Plate13-Degenerate AF-donor13
Plate14-Degenerate AF-donor13
NC1 NC2 NC3 NC4 NC5 NPC1 NPC2 NPC3 AF-O AF-I AF-VEC INF
g
Adult NP-donor7
Adult NP-donor8
Adult NP-donor9
Degenerate NP-donor13
Degenerate NP-donor14
Degenerate NP-donor15
Degenerate AF-donor13
h
H&E
S-O/FG
50 μm
i
300 μm
50 μm
j
spliced
unspliced
ambiguous
Cluster
proportions

Fig. S4 Application of single-cell T-ChIC to study transcriptome and H3K27me3 dynamics in disc cells of NCD dogs. **a** Schematic of the methodology. Fixed, permeabilized cells are stained with an antibody targeting H3K27me3. Inactive pA-MN is added to tether MN to the histone modification antibody. Single cells are FACS-sorted, whereafter MN activation induces specific cuts in the genome. UMIs and cell-specific barcodes are ligated to the cut fragments. Barcoded fragments are pooled, amplified and sequenced. Additionally, full-length RNA is retrieved following RNA fragmentation, repair and A tailing. **b-d** Dog cells were mixed with human K562 cells to maintain a constant antibody to cell ratio, whereafter K562 cells were removed during FACS by CellTrace Far Red staining. Dead dog cells were removed by Zombie red staining; the recovered viable cells were quantified and collected with consistency when using the swing bucket rotor. **e** Transcript-based UMAP coloured according to the scanpy score_genes combined marker score[41] for identification of NC, NPC, INF, AF-I, AF-O and AF-VECS clusters. **f-g** Contribution of donor source and sequencing run to each cluster. Cells annotated by sample origin (N=3 adult NC-rich and N=3 degenerate discs obtained during standard-of-care surgery). The degenerate annulus fibrosus (AF sample) was from donor 13. The detailed donor information is shown in Table S1. **h-i** Hematoxylin and eosin (H&E), Safranin-O/Fast green (S-O/FG) and collagen type 1 (COL1) stained tissue sections from patient donor 13, containing NP tissue herniated into the AF tissue, reveal scattered vacuolated NCs alongside NPCs. Arrow denote NCs and triangle indicate NPCs. A zoom (black box) highlights the characteristic vacuolated NC morphology. **j** RNA splicing statistics showing the proportions of spliced/unspliced counts per cluster. T-ChIC typically yields ~60% of unspliced molecules containing intronic sequences.

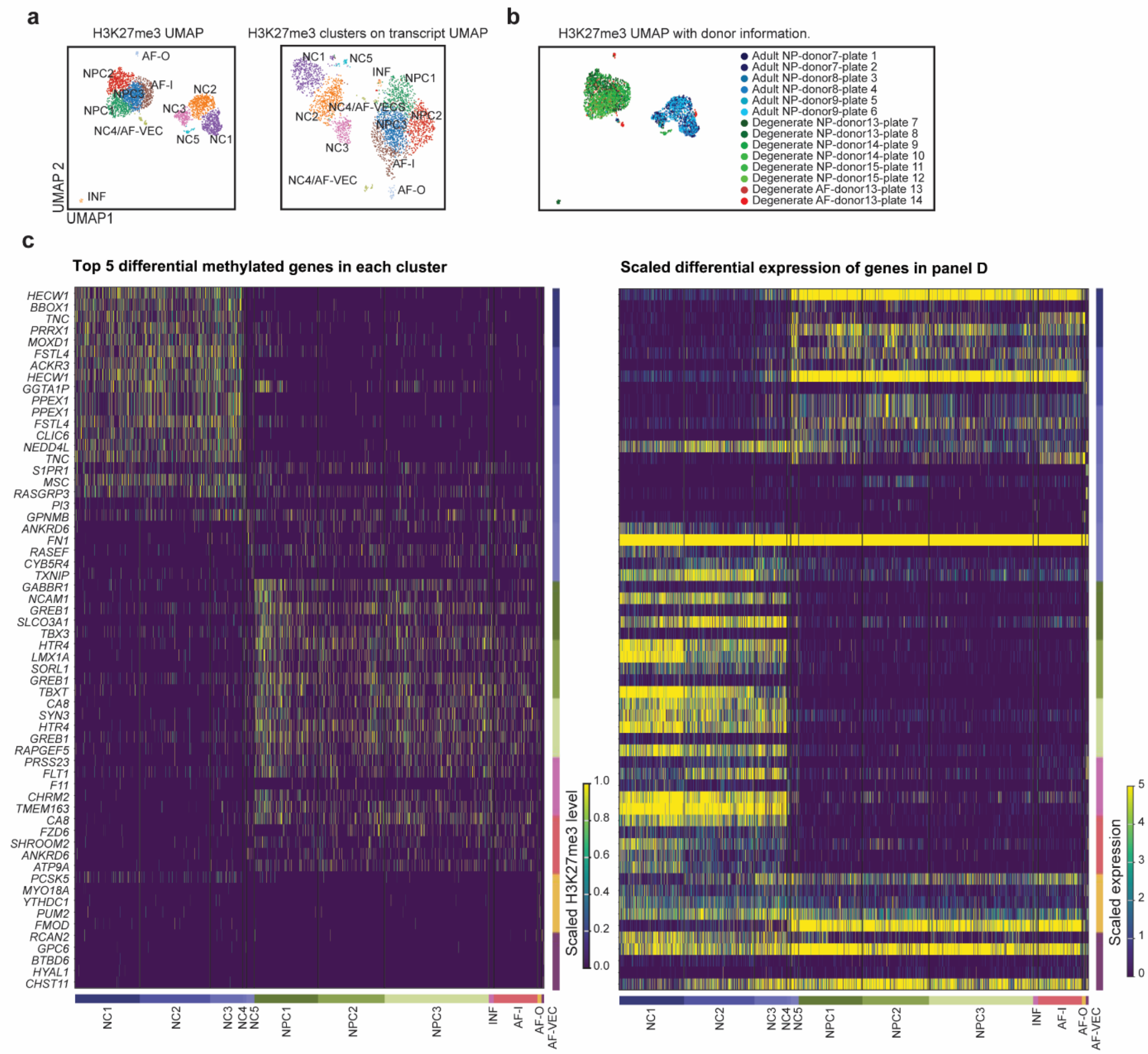
a
H3K27me3 UMAP
AF-O
NPC2
AF-I
NC2
NPC3
NPC1
NC3
NC4/AF-VEC
NC5
NC1
INF
UMAP 2
UMAP1
H3K27me3 clusters on transcript UMAP
NC1
NC5
INF
NPC1
NC4/AF-VECS
NC2
NPC2
NPC3
NC3
AF-I
NC4/AF-VEC
AF-O
b
H3K27me3 UMAP with donor information.
Adult NP-donor7-plate 1
Adult NP-donor7-plate 2
Adult NP-donor8-plate 3
Adult NP-donor8-plate 4
Adult NP-donor9-plate 5
Adult NP-donor9-plate 6
Degenerate NP-donor13-plate 7
Degenerate NP-donor13-plate 8
Degenerate NP-donor14-plate 9
Degenerate NP-donor14-plate 10
Degenerate NP-donor15-plate 11
Degenerate NP-donor15-plate 12
Degenerate AF-donor13-plate 13
Degenerate AF-donor13-plate 14
c
Top 5 differential methylated genes in each cluster
Scaled differential expression of genes in panel D
HECW1
BBOX1
TNC
PRRX1
MOXD1
FSTL4
ACKR3
HECW1
GGTA1P
PPEX1
PPEX1
FSTL4
CLIC6
NEDD4L
TNC
S1PR1
MSC
RASGRP3
PI3
GPNMB
ANKRD6
FN1
RASEF
CYB5R4
TXNIP
GABBR1
NCAM1
GREB1
SLCO3A1
TBX3
HTR4
LMX1A
SORL1
GREB1
TBXT
CA8
SYN3
HTR4
GREB1
RAPGEF5
PRSS23
FLT1
F11
CHRM2
TMEM163
CA8
FZD6
SHROOM2
ANKRD6
ATP9A
PCSK5
MYO18A
YTHDC1
PUM2
FMOD
RCAN2
GPC6
BTBD6
HYAL1
CHST11
Scaled H3K27me3 level
1.0
0.8
0.6
0.4
0.2
0.0
Scaled expression
5
4
3
2
1
0
NC1
NC2
NC3
NC4
NC5
NPC1
NPC2
NPC3
INF
AF-I
AF-O
AF-VEC

d

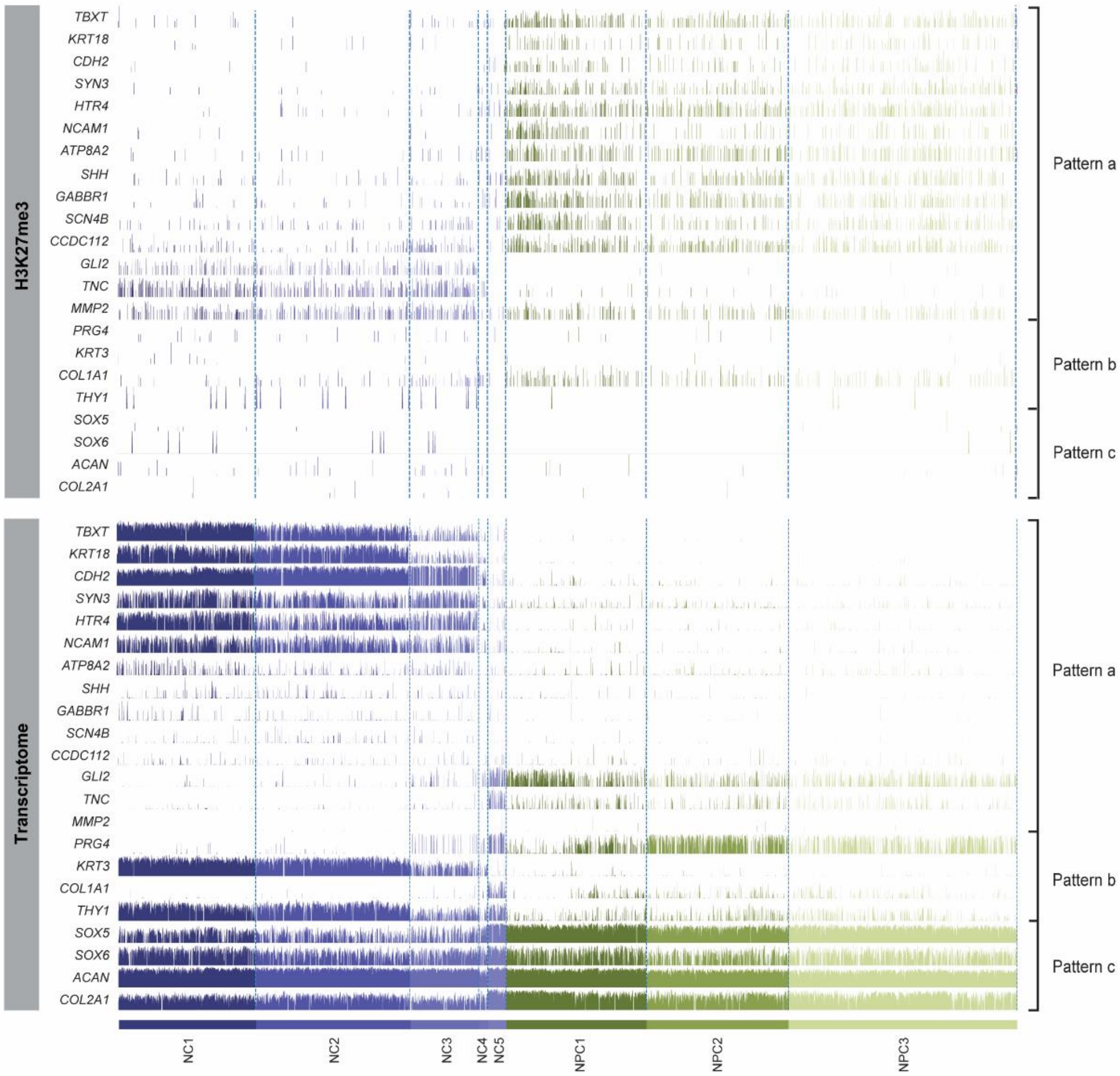
H3K27me3
TBXT
KRT18
CDH2
SYN3
HTR4
NCAM1
ATP8A2
SHH
GABBR1
SCN4B
CCDC112
GLI2
TNC
MMP2
PRG4
KRT3
COL1A1
THY1
SOX5
SOX6
ACAN
COL2A1
Pattern a
Pattern b
Pattern c
Transcriptome
NC1
NC2
NC3
NC4
NC5
NPC1
NPC2
NPC3

e

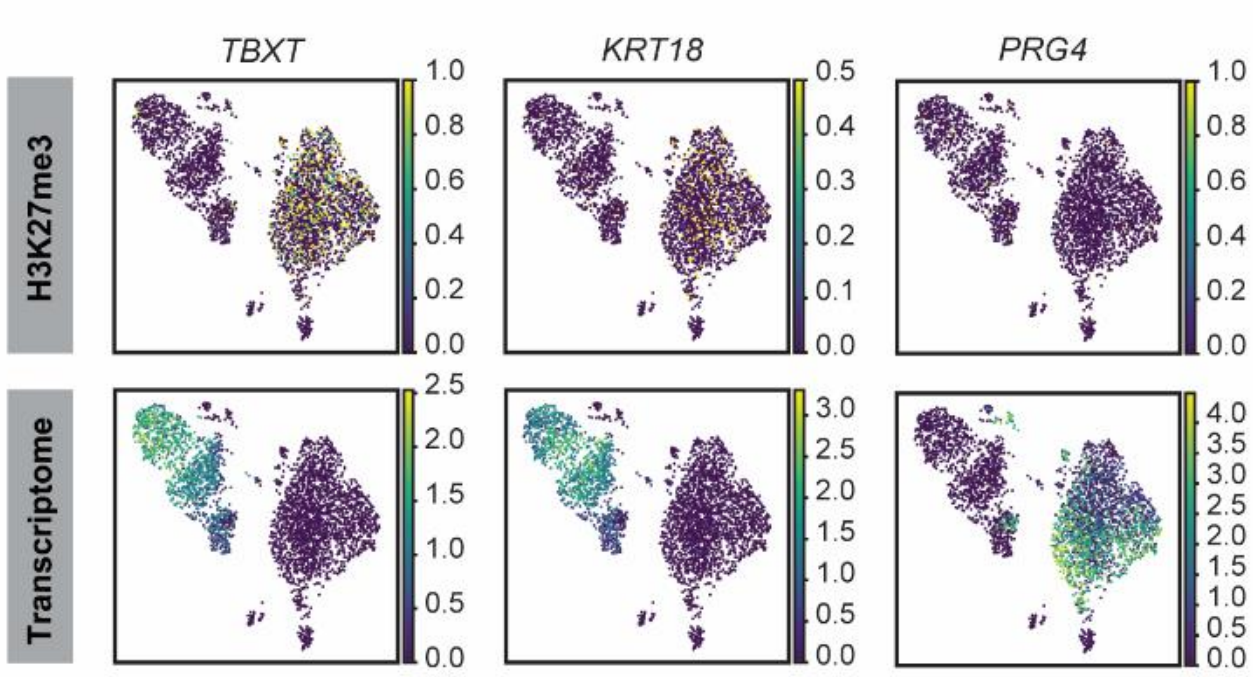
TBXT
KRT18
PRG4
H3K27me3
Transcriptome

Fig. S5 Differential H3K27me3 enrichment reveals epigenetic regulation of NC to NPC fate transition. **a-b** H3K27me3-based UMAP, with 11 clusters identified by unsupervised clustering and donor contribution. **c** Heatmaps of the top 5 differentially methylated genes per cluster, sorted by average log fold change, with corresponding scaled expression, highlighting distinct patterns of H3K27me3 and gene expression defining NC and non-NC cell populations. Cluster colours are specific to the single-cell T-ChIC data set and do not correspond to those used for the single cell RNAseq dataset presented through Figures 1-3. **d** Full-length RNA and H3K27me3 track plots of key NC genes revealing three main patterns: (a) H3K27me3 enrichment with gene silencing, (b) differential expression independent of H3K27me3, and (c) Genes that are consistently expressed, with no evidence of H3K27me3-mediated silencing observed in this study. **e** Transcript-based UMAPs coloured by RNA and H3K27me3 enrichment for selected genes. The scalebar with gradient colour represent the intensity of the enrichments.

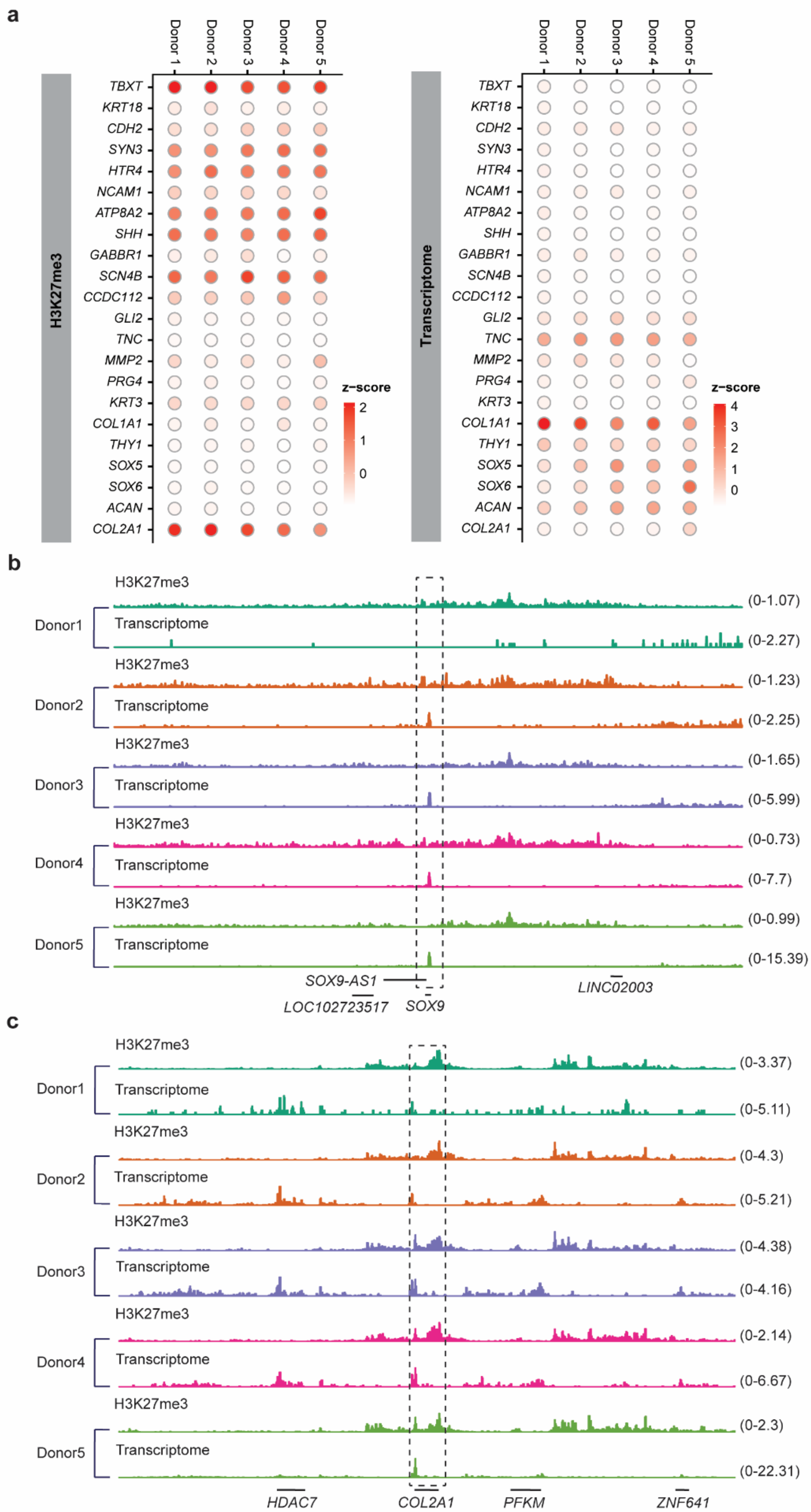

a
H3K27me3
Transcriptome
Donor 1
Donor 2
Donor 3
Donor 4
Donor 5
TBXT
KRT18
CDH2
SYN3
HTR4
NCAM1
ATP8A2
SHH
GABBR1
SCN4B
CCDC112
GLI2
TNC
MMP2
PRG4
KRT3
COL1A1
THY1
SOX5
SOX6
ACAN
COL2A1
z-score
b
Donor1
Donor2
Donor3
Donor4
Donor5
(0-1.07)
(0-2.27)
(0-1.23)
(0-2.25)
(0-1.65)
(0-5.99)
(0-0.73)
(0-7.7)
(0-0.99)
(0-15.39)
SOX9-AS1
LOC102723517
SOX9
LINC02003
c
(0-3.37)
(0-5.11)
(0-4.3)
(0-5.21)
(0-4.38)
(0-4.16)
(0-2.14)
(0-6.67)
(0-2.3)
(0-22.31)
HDAC7
COL2A1
PFKM
ZNF641

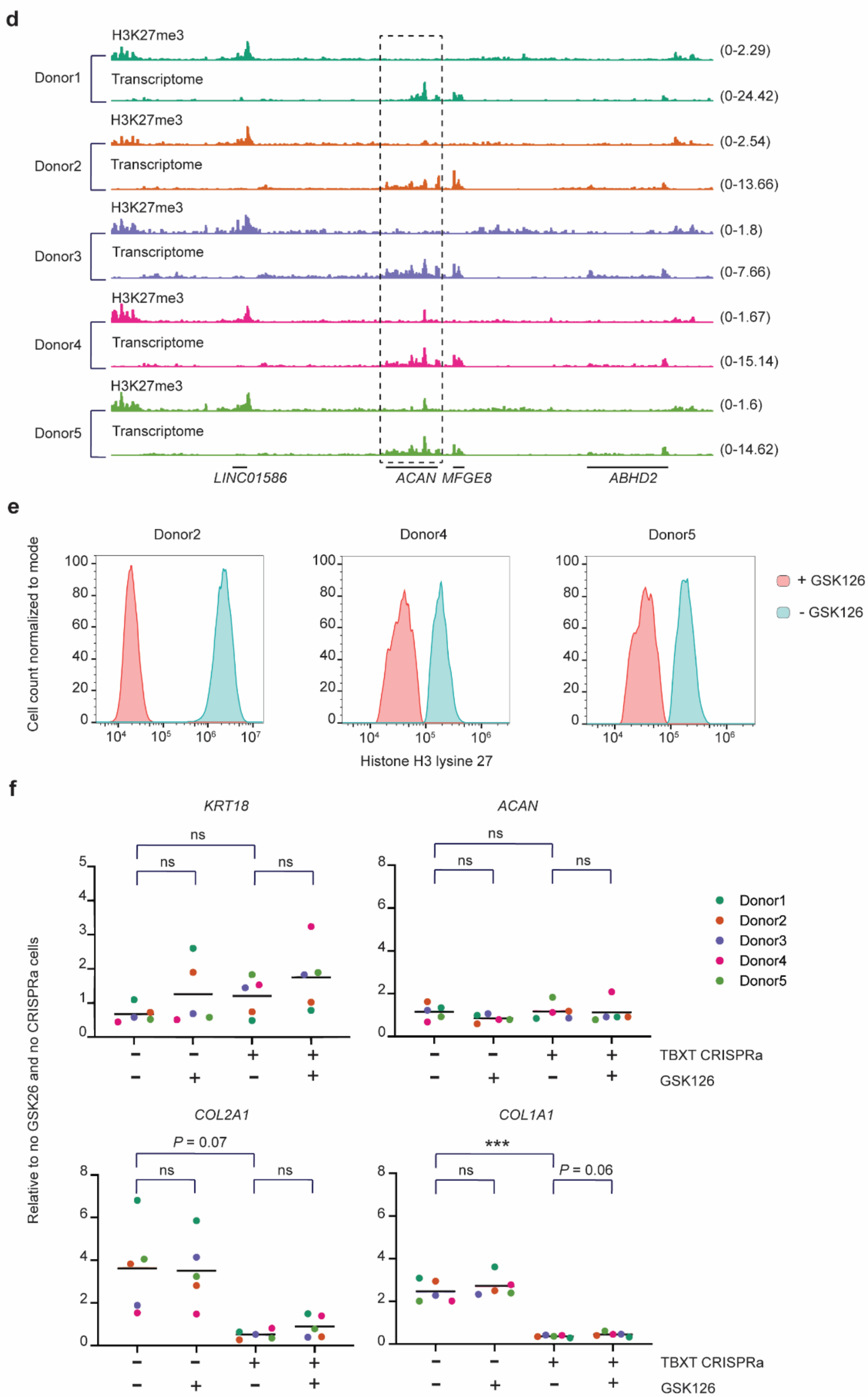
d
H3K27me3
Transcriptome
Donor1
Donor2
Donor3
Donor4
Donor5
(0-2.29)
(0-24.42)
(0-2.54)
(0-13.66)
(0-1.8)
(0-7.66)
(0-1.67)
(0-15.14)
(0-1.6)
(0-14.62)
LINC01586
ACAN
MFGE8
ABHD2
e
Donor2
Donor4
Donor5
Cell count normalized to mode
Histone H3 lysine 27
+ GSK126
- GSK126
f
KRT18
ACAN
COL2A1
COL1A1
ns
P = 0.07
P = 0.06
***
Donor1
Donor2
Donor3
Donor4
Donor5
TBXT CRISPRa
GSK126
Relative to no GSK26 and no CRISPRa cells

Fig. S6 Methyl transferase inhibition of human NPCs to reverse their degenerative phenotype. **a** Scaled dot plots showing the normalised read counts for H3K27me3 enrichment and gene expression of key NC genes for each donor. **b-d** T-ChIC profiles from five human NPC donors showing localized H3K27me3 enrichment. The detailed H3K27me3 and transcriptome profiles are presented in Data file S4 and S5. **e** Flow cytometry showing H3K27me3 loss after GSK126 treatment in donor 2, 4, and 5. The staining for donor 3 failed due to technical reasons and could not be repeated. **f** Gene expression following GSK126 treatment and combined GSK126 + CRISPRa activation. *** $p < 0.001$

Table S1: Sample details and grading of dog IVDs used in the study

Donor	Source	Breed	Sample	Sex	Age	clinical diagnosis	disc level	Deg. grade	sort seq/ t-chic plate
**1**	Still born	LR	NP	UD	Stillborn	NA	C1-L5	H	scRNA-seq Plate 1
**2**	Still born	LR	NP	UD	Stillborn	NA	C1-L5	H	scRNA-seq Plate 2
**3**	Still born	LR	NP	UD	Stillborn	NA	C1-L5	H	scRNA-seq Plate 3
**4**	Adult	MON	NP	F	13 mo	NA	C1-L5		scRNA-seq Plate 4, 5
**5**	Adult	MON	NP	M	17 mo	NA	C1-L5	H	scRNA-seq Plate 6
**6**	Adult	MON	NP	M	16 mo	NA	C1-L5	H	scRNA-seq Plate 7
**7**	Adult	MON	NP	M	14 mo	NA	C1-L5	H	T-ChIC-seq Plate 1, 2
**8**	Adult	MON	NP	M	14 mo	NA	C1-L5	H	T-ChIC-seq Plate 3, 4
**9**	Adult	MON	NP	M	14 mo	NA	C1-L5	H	T-ChIC-seq Plate 5, 6
**10**	Patient	BM	NP	F	7.5 y	DLSS	L7-S1	SD	scRNA-seq Plate 8
**11**	Patient	LR	NP	F	6.5 y	DLSS	L7-S1	SD	scRNA-seq Plate 9
**12**	Patient*	BM	NP	M	6 y	DLSS	L7-S1	SD	scRNA-seq Plate 10
**12**	Patient*	BM	AF	M	6 y	DLSS	L7-S1	MI-D	scRNA-seq Plate 11
**13**	Patient#	RR	NP	M	9.8 y	DLSS	L7-S1	SD	T-ChIC-seq Plate 7, 8
**14**	Patient	GR	NP	F	10 y	DLSS	L7-S1	SD	T-ChIC-seq Plate 9, 10
**15**	Patient	BS	NP	F	8 y	DLSS	L7-S1	SD	T-ChIC-seq Plate 11, 12
**13**	Patient#	RR	AF	M	9.8 y	DLSS	L7-S1	SD	T-ChIC-seq Plate 13, 14

Note: An experienced veterinary pathologist scored the degeneration grade, and the breakdown of parameters used to histologically score degeneration is indicated in Table S2 to S3. H = healthy, MI-D = mildly degenerated, MO-D = moderately degenerated and SD = severely degenerated. *Note sample 12 and 13 are from the same donor, where NP and AF from surgical samples were used in the RNA-seq analysis. #Note sample 14 and 17 are from the same donor, where NP and AF from surgical samples were used in the RNA-seq analysis. LR, Labrador Retriever; MON, Mongrel; BM, Bernese Mountain dog; RR, Rhodesian Ridgeback; GR, Golden Retriever; BS, Belgian Shepherd dog; DLSS, Degenerative lumbosacral stenosis; UD, undocumented, NP, nucleus pulposus; AF, annulus fibrosus; NA, not applicable; mo: months; y: years; F: female; M: male.

Table S2: Breakdown of the parameters used for scoring degeneration of NP

CRITERIUM		SCORE
**1**	**Presence of notochordal cells (NCs) in the NP (H&E)**	
	Abundantly present (>90% of total cells) within the entire NP	0
	Present in moderate amounts (50-90% of total cells)	1
	Present in low amounts (<50% of total cells)	2
	Absent	3
**2**	**Nucleus pulposus cell (NPC) clusters (H&E)**	
	No clusters	0
	Scattered small cell clusters (i.e., 2-7 nuclei per cluster)	1
	Frequent large cell clusters (i.e., >8 nuclei per cluster)	2
	Presence of huge cell clusters (i.e., >15 nuclei per cluster)	3
**3**	**NP cell loss (karyolysis) and cell necrosis (pyknosis or karyorrhexis) (H&E)**	
	None - lacunae all contain viable nuclei	0
	Rarely present. Few (<25%) lacunae demonstrate karyolysis, pyknosis, or karyorrhexis	1
	Frequently present. 25%-50% of lacunae demonstrate karyolysis, pyknosis, or karyorrhexis	2
	Karyolysis, pyknosis, or karyorrhexis predominates (>50%)	3
**4**	**NP Matrix staining (AB-PSR)**	
	Blue proteoglycan ECM stain dominates	0
	Reduction in blue proteoglycan ECM staining (i.e., fading)	1
	Reduction in blue proteoglycan ECM staining with presence of collagen staining (up to 50% of area)	2
	Loss of blue proteoglycan ECM staining and/or dominance of collagen staining (>50% of area)	3

Note: For grading, the scores are summed up and divided by 4. Grades have been assigned according to the criteria outlined, 0-0.4, healthy; 0.5-1.4, mildly degenerated; 1.5-2.4, moderately degenerated; ≥2.5, severely degenerated. This grading table is adapted from the study by Lee et al.[3]

Table S3: Breakdown of the parameters used for scoring degeneration of AF

CRITERIUM		SCORE
**1**	**AF morphology (AB-PSR)**	
	Well-organized, well-defined, uniform collagen lamellae form concentric half-ring arcs throughout entire AF	0
	Mild disorganization/delamination of collagen fiber lamellae with some disruption or loss of concentric layers (<25%)	1
	Moderately disorganization/delamination of collagen fiber lamellae with progressive disruption or loss of concentric layer (25–75%)	2
	Complete disorganization/delamination/collapse of AF; almost all concentric collagen lamellae are severely disrupted or lost (>75%)	3
**2**	**AF Cellular (H&E) and ECM metaplasia (AB-PSR)**	
	Spindle-shaped fibrocytic nuclei populate AF with no or rare individual metaplastic cells resembling NPCs in the AF	0
	Proliferation of cells resembling NPC's is focally restricted (i.e. perilesional) or mild proliferation with >75% lacunae containing single fibrocytic nuclei (i.e. rare to few small NPC-like clusters)	1
	Regionally extensive perilesional proliferation of cells resembling NPC's or moderate proliferation involving 25-50% AF lacunae that contain more than one cell nucleus (i.e. frequent NPC-like clusters).	2
	Regionally extensive perilesional proliferation of cells resembling NPC's or moderate proliferation involving >50% AF lacunae that contain more than one cell nucleus (i.e. frequent NPC-like clusters).	3

Note: For grading, the scores are summed up and divided by 2. Grades have been assigned according to the criteria outlined, 0-0.4, healthy; 0.5-1.4, mildly degenerated; 1.5-2.4, moderately degenerated; ≥2.5, severely degenerated. Note that the question on tears and cleft formation (AB-PSR and H&E) was left out because of fragmentation of the tissue during surgical removal. This grading table is adapted from the study by Lee *et al*.[3]

Table S4: Cells per cluster per tissue type and per cell donor in the SORT-Seq data set

Donor	J-NC1	J-NC2	J-NC3	M-NC1	M-NC2	M-NC3	M-NC4	NPC	AF-I	AF-O	AF-VECs	IVDD-F	INF
Plate 1-Juvenile NP-donor1	154	33	35	7	0	0	0	0	0	0	0	0	0
Plate 2-Juvenile NP-donor2	44	111	114	3	0	0	0	0	0	0	0	0	0
Plate 3-Juvenile NP-donor3	61	199	59	5	0	0	0	0	0	0	0	0	0
Plate 4-Adult NP-donor4	0	0	0	60	15	129	1	0	0	0	0	0	0
Plate 5-Adult NP-donor4	0	1	0	45	25	124	3	0	0	0	0	0	0
Plate 6-Adult NP-donor5	0	1	0	54	26	122	7	11	4	3	0	0	0
Plate 7-Adult NP-donor6	0	4	0	55	27	84	24	23	3	0	0	4	0
Plate 8-Degenerate NP-donor 10	0	2	0	2	0	0	0	17	8	12	0	13	7
Plate 9-Degenerate NP-donor 11	0	0	0	1	0	0	2	289	2	0	0	2	1
Plate 10-Degenerate NP-donor 12	0	0	0	3	0	0	0	0	62	7	0	192	10
Plate 11-Degenerate AF-donor 12	0	0	0	3	1	0	0	1	18	180	41	15	26

Table S5: Cells per cluster per tissue type and per cell donor in the T-ChIC data set

Donor	NC 1	NC 2	NC 3	NC 4	NC 5	NPC 1	NPC 2	NPC 3	IN F	AF-I	AF-O	AF-VEC	ALL
Plate 1,2-Adult NP-donor7	181	147	77	9	4	2	0	3	0	1	0	0	424
Plate 3,4-Adult NP-donor8	152	144	39	2	2	3	0	0	0	0	0	0	342
Plate 5,6-Adult NP-donor9	111	204	71	3	0	2	0	0	0	0	0	0	391
Plate 7,8-Degenerate NP-donor13	0	0	36	3	6	66	257	210	22	87	2	0	689
Plate9,10-Degenerate NP-donor14	0	0	0	1	24	85	109	135	0	160	1	0	515
Plate 11,12-Degenerate NP-donor15	1	5	0	7	0	202	12	194	5	43	0	0	469
Plate 13,14-Degenerate AF-donor13	0	0	0	4	23	95	83	199	7	39	56	41	547

Table S6: Donor information

Donor	Age	Gender	Tissue	Thompson scoring
**1**	81	Female	Thoracic disc	IV
**2**	74	Female	Cervical disc	not available*
**3**	89	Male	Lumbar disc	IV
**4**	64	Male	Lumbar disc	V
**5**	58	Male	Lumbar disc	IV

(*): the Thompson grade for donor 2 was not noted for the cervical discs when tissues were collected to isolate human NPCs. The lumbar discs were collected for an unrelated experiment and were graded grade V.

Table S7: List of qPCR primers

Genes	Forward sequence	Reverse sequence	Tm (°C)
TBXT	CCTGAGACCCAGTTCATAGC	GTTCCTCCATCATCTCTTTGTG	64
SOX9	CAAGCTCTGGAGACTTCTGAACG	CCGTTCTTCACCGACTTCCT	60
KRT18	CGGGCATTGTCCACAGTATT	GGGAGCACTTGGAGAAGAAG	65
ACAN	CAACTACCCGGCCATCC	GATGGCTCTGTAATGGAACAC	64
COL2A1	AGGGCCAGGATGTCCGGCA	GGGTCCCAGGTTCTCCATCT	63
COL1A1	TCCAACGAGATCGAGATCC	AAGCCGAATTCCTGGTCT	60
GAPDH	CAAGATCATCAGCAATGCCT	CAGGGATGATGTTCTGGAGAG	60
RPS5	TGCAGGATTACATTGCAGTG	CATCATGGAGTTAGTGAGGC	60

Table S8: Immunohistochemical staining

Antibody	Catalog no.	Dilution	Antigen retrieval	host	block	Secondary antibody	Detection system	Isotype control
Sox9	AB5535, Merck Millipore	1:250	none	Rabbit	1. 0,3% H_2O_2 (Sigma Aldrich, 30%w/w), RT,10min 2. NHS 5%+NGS 5% in PBS, RT, 30min	Brightvision Anti-Rabbit IgG, HRP conjugated	BrightDAB, substrate DAB (Immunologic)	Rabbit Immunoglobulin Fraction, DAKO X0903, 20 mg/mL
Col1	AB6308, Abcam	1:1.500	1.Pronase;37ºC, 30min 2. Hyaluronidase; 37 ºC, 30min	Mouse	1. 0,3% H_2O_2 (Sigma Aldrich, 30%w/w), RT,10min 2. PBS-BSA 5%, RT, 30min	BrightVision Poly HRP anti-Mouse IgG (Immunologic VWRKDPVM110 HRP)	BrightDAB, substrate DAB (Immunologic)	Mouse IgG1 (DAKO X0931, 100 mg/ML)
TBXT	AF2085, R&D systems	1:50	10 mM Citrate buffer, pH 6, 70ºc, 30min	Goat	1. 0,3% H_2O_2 (Sigma Aldrich, 30%w/w), RT, 5min 2. NHS-PBS 10%, RT, 30min	Horse Anti-Goat IgG Antibody (H+L), Biotinylated (BA-9500-1.5)+ VECTASTAIN® ABC-HRP Kit (vector laboratories)	BrightDAB, substrate DAB (Immunologic)	Goat IgG, R&D Systems, AF496 1mg/mL
IBA-1	MABN92, Merck	1:2.500	10 mM Citrate buffer, pH 6, 70ºc, 30min	Mouse	1. 0,3% H_2O_2 (Sigma Aldrich, 30%w/w), RT,10min 2. PBS-BSA 5%+ 10% NGS RT, 15min	BrightVision Poly HRP anti-Mouse IgG (Immunologic VWRKDPVM110 HRP)	BrightDAB, substrate DAB (Immunologic)	Mouse IgGk, Abcam, AB91353 0.1mg/mL

NHS: normal horse serum, NGS:normal goat serum, BSA:bovine serum albumin.

References Supplementary File –

1 Williams, R. J. *et al.* Recommendations for intervertebral disc notochordal cell investigation: From isolation to characterization. *JOR Spine* **6**, e1272 (2023). https://doi.org/10.1002/jsp2.1272

2 Worth, A., Meij, B. & Jeffery, N. Canine degenerative lumbosacral stenosis: prevalence, impact and management strategies. *Veterinary Medicine: research and reports*, 169-183 (2019).

3 Lee, N. N. *et al.* A comprehensive tool box for large animal studies of intervertebral disc degeneration. *JOR Spine* **4**, e1162 (2021). https://doi.org/10.1002/jsp2.1162

4 Burckstummer, T. *et al.* A reversible gene trap collection empowers haploid genetics in human cells. *Nat Methods* **10**, 965-971 (2013). https://doi.org/10.1038/nmeth.2609

5 Muraro, M. J. *et al.* A Single-Cell Transcriptome Atlas of the Human Pancreas. *Cell Syst* **3**, 385-394 e383 (2016). https://doi.org/10.1016/j.cels.2016.09.002

6 Hashimshony, T. *et al.* CEL-Seq2: sensitive highly-multiplexed single-cell RNA-Seq. *Genome biology* **17**, 1-7 (2016).

7 Hornung, B. V. *et al.* Comparison of single cell transcriptome sequencing methods: of mice and men. *Genes* **14**, 2226 (2023).

8 Parekh, S., Ziegenhain, C., Vieth, B., Enard, W. & Hellmann, I. zUMIs-A fast and flexible pipeline to process RNA sequencing data with UMIs. *Gigascience* **7**, giy059 (2018).

9 Hao, Y. *et al.* Integrated analysis of multimodal single-cell data. *Cell* **184**, 3573-3587. e3529 (2021).

10 Choudhary, S. & Satija, R. Comparison and evaluation of statistical error models for scRNA-seq. *Genome biology* **23**, 27 (2022).

11 Andreatta, M. & Carmona, S. J. UCell: Robust and scalable single-cell gene signature scoring. *Computational and structural biotechnology journal* **19**, 3796-3798 (2021).

12 Pachkov, M., Balwierz, P. J., Arnold, P., Ozonov, E. & Van Nimwegen, E. SwissRegulon, a database of genome-wide annotations of regulatory sites: recent updates. *Nucleic acids research* **41**, D214-D220 (2012).

13 Street, K. *et al.* Slingshot: cell lineage and pseudotime inference for single-cell transcriptomics. *BMC Genomics* **19**, 477 (2018). https://doi.org/10.1186/s12864-018-4772-0

14 Salmen, F. *et al.* High-throughput total RNA sequencing in single cells using VASA-seq. *Nat Biotechnol* **40**, 1780-1793 (2022). https://doi.org/10.1038/s41587-022-01361-8

15 Zeller, P. *et al.* Single-cell sortChIC identifies hierarchical chromatin dynamics during hematopoiesis. *Nat Genet* **55**, 333-345 (2023). https://doi.org/10.1038/s41588-022-01260-3

16 Blotenburg, M. *et al.* Detection of histone modifications and full-length transcriptome in single cells identifies sequential epigenetic perceptiveness to lineage commitment. *bioRxiv*, 2024.2005. 2009.593364 (2024).

17 Cai, J. *et al.* Assessing self-renewal and differentiation in human embryonic stem cell lines. *Stem cells* **24**, 516-530 (2006).
18 Schmid, M., Durussel, T. & Laemmli, U. K. ChIC and ChEC: genomic mapping of chromatin proteins. *Molecular cell* **16**, 147-157 (2004).
19 Weiler, P., Van den Berge, K., Street, K. & Tiberi, S. in *Single Cell Transcriptomics: Methods and Protocols* 269-292 (Springer, 2022).
20 Thul, P. J. *et al.* A subcellular map of the human proteome. *Science* **356** (2017). https://doi.org/10.1126/science.aal3321
21 Hansen, K. H. *et al.* A model for transmission of the H3K27me3 epigenetic mark. *Nature cell biology* **10**, 1291-1300 (2008).
22 McCabe, M. T. *et al.* EZH2 inhibition as a therapeutic strategy for lymphoma with EZH2-activating mutations. *Nature* **492**, 108-112 (2012).
23 Kim, J. *et al.* Polycomb-and methylation-independent roles of EZH2 as a transcription activator. *Cell reports* **25**, 2808-2820. e2804 (2018).
24 Rao, R. A. *et al.* Ezh2 mediated H3K27me3 activity facilitates somatic transition during human pluripotent reprogramming. *Scientific reports* **5**, 8229 (2015).
25 Konermann, S. *et al.* Genome-scale transcriptional activation by an engineered CRISPR-Cas9 complex. *Nature* **517**, 583-588 (2015).
26 Wu, Y. *et al.* Leveraging CRISPR activation for rapid assessment of gene editing products in human pluripotent stem cells. *Stem Cell Reports* (2025).
27 Bach, F. C. *et al.* Biologic canine and human intervertebral disc repair by notochordal cell-derived matrix: from bench towards bedside. *Oncotarget* **9**, 26507-26526 (2018). https://doi.org/10.18632/oncotarget.25476
28 Dabin, J., Fortuny, A. & Polo, S. E. Epigenome Maintenance in Response to DNA Damage. *Mol Cell* **62**, 712-727 (2016). https://doi.org/10.1016/j.molcel.2016.04.006
29 Kamali, S. A. *et al.* High-intensity zones in dogs with lumbosacral intervertebral disc degeneration: insights from MRI and histopathological findings. *Veterinary Quarterly* **45**, 1-13 (2025).
30 Adkar, S. S. *et al.* Step-Wise Chondrogenesis of Human Induced Pluripotent Stem Cells and Purification Via a Reporter Allele Generated by CRISPR-Cas9 Genome Editing. *Stem Cells* **37**, 65-76 (2019). https://doi.org/10.1002/stem.2931
31 Warin, J. *et al.* In vitro and in vivo models define a molecular signature reference for human embryonic notochordal cells. *Iscience* **27**, 109018 (2024).
32 Duff, S. E., Li, C., Garland, J. M. & Kumar, S. CD105 is important for angiogenesis: evidence and potential applications. *The FASEB Journal* **17**, 984-992 (2003).
33 Sarmah, S., Hawkins, M. R., Manikandan, P., Farrell, M. & Marrs, J. A. Elf3 deficiency during zebrafish development alters extracellular matrix organization and disrupts tissue morphogenesis. *PLoS One* **17**, e0276255 (2022).

34 José-Edwards, D. S. *et al.* The identification of transcription factors expressed in the notochord of Ciona intestinalis adds new potential players to the brachyury gene regulatory network. *Developmental Dynamics* **240**, 1793-1805 (2011).

35 Yao, J. *et al.* Cloning and developmental expression of the DEC1 ortholog gene in zebrafish. *Gene expression patterns* **6**, 919-927 (2006).

36 La Spina, M. *et al.* MiT/TFE family of transcription factors: an evolutionary perspective. *Frontiers in Cell and Developmental Biology* **8**, 609683 (2021).

37 Sperber, S. M. & Dawid, I. B. barx1 is necessary for ectomesenchyme proliferation and osteochondroprogenitor condensation in the zebrafish pharyngeal arches. *Developmental biology* **321**, 101-110 (2008).

38 Kubra, K. *et al.* Phylogenetic and expression analysis of Fos transcription factors in zebrafish. *International Journal of Molecular Sciences* **23**, 10098 (2022).

39 He, X., Ohba, S., Hojo, H. & McMahon, A. P. AP-1 family members act with Sox9 to promote chondrocyte hypertrophy. *Development* **143**, 3012-3023 (2016).

40 Naba, A. *et al.* The matrisome: in silico definition and in vivo characterization by proteomics of normal and tumor extracellular matrices. *Molecular & Cellular Proteomics* **11** (2012).

41 Wolf, F. A., Angerer, P. & Theis, F. J. SCANPY: large-scale single-cell gene expression data analysis. *Genome biology* **19**, 1-5 (2018).
